# Successful treatment of severe sepsis and diarrhea after vagotomy utilizing fecal microbiota transplantation: a case report

**DOI:** 10.1186/s13054-015-0738-7

**Published:** 2015-02-09

**Authors:** Qiurong Li, Chenyang Wang, Chun Tang, Qin He, Xiaofan Zhao, Ning Li, Jieshou Li

**Affiliations:** Research Institute of General Surgery, Jinling Hospital, Nanjing University School of Medicine, No.305 East Zhongshan Road, Nanjing, 210002 China

## Abstract

**Introduction:**

Dysbiosis of intestinal microbiota likely plays an important role in the development of gut-derived infections, making it a potential therapeutic target against sepsis. However, experience with fecal microbiota transplantation (FMT) in the treatment of sepsis and knowledge of the underlying mechanisms are extremely lacking. In this article, we describe a case of a patient who developed sepsis after a vagotomy and later received an infusion of donor feces microbiota, and we report our findings.

**Methods:**

A 44-year-old woman developed septic shock and severe watery diarrhea 4 days after undergoing a vagotomy. Antibiotics, probiotics and supportive treatment strategies were used for about 30 day after surgery, but the patient’s fever, bacteremia and watery diarrhea persisted. Considering the possibility of intestinal dysbiosis, we evaluated the structure and composition of the patient’s fecal microbiota using 16S rDNA-based molecular techniques. As expected, the gut microbiota was extensively disrupted; therefore, a donor fecal suspension was delivered into the patient by nasoduodenal tube. The patient’s clinical outcomes and shifts of the gut microbiota following the treatment were also determined.

**Results:**

Dramatically, the patient’s septic symptoms and severe diarrhea were successfully controlled following FMT. Her stool output markedly declined after 7 days and normalized 16 days after FMT. A significant modification in her microbiota composition was consistently seen, characterized by a profound enrichment of the commensals in Firmicutes and depletion of opportunistic organisms in Proteobacteria. Furthermore, we identified a reconstituted bacterial community enriched in Firmicutes and depleted of Proteobacteria members that was associated with fecal output, plasma markers of inflammation and T helper cells.

**Conclusions:**

In this report, we describe our initial experience with FMT, in which we successfully used it in the treatment of a patient with sepsis and severe diarrhea after a vagotomy. Our data indicate an association between repaired intestinal microbiota barrier and improvement of clinical outcomes. Our patient’s surprising clinical benefits from FMT demonstrate the role of intestinal microbiota in modulating immune equilibrium. It represents a breakthrough in the clinical management of sepsis and suggests new therapeutic avenues to pursue for microbiota-related indications.

**Electronic supplementary material:**

The online version of this article (doi:10.1186/s13054-015-0738-7) contains supplementary material, which is available to authorized users.

## Introduction

The mucosal surface of the gastrointestinal tract is colonized by a complex ecosystem of commensal microbiota that mediates homeostatic effects on the host and shapes aspects of host metabolism, immune functions and protection against invasion by pathogens [1-3]. It is now well appreciated that the intestinal microbiota constitutes an efficient microbial barrier against infections and is critical to the host antimicrobial defense. Protracted loss of the typical microbiota composition has been associated with exposure to antibiotics, inflammation and several disorders, including inflammatory bowel disease [4,5]. Recurrent *Clostridium difficile* infection (CDI) is thought to result from persistent disruption of commensal gut microbiota [6]. The reestablishment of intestinal microbiota balance is needed in a curative approach for therapy. Recently, fecal microbiota transplantation (FMT) has emerged as a critical treatment for recurrent CDI [7,8]. However, whether an ecologically stable microbial population is restored and the nature of the transition remain to be elucidated.

Sepsis is one of the leading causes of mortality in the intensive care unit (ICU), with rates of approximately 50% to 60% in patients who develop septic shock and 30% to 50% in those who develop severe sepsis [9,10]. Therapy for severe sepsis is still largely supportive and based on symptoms. The commensal enteric microbiota constitutes a pivotal microbial barrier that protects against opportunistic pathogen invasion [1-3]. The gut microbiota is essential for the maintenance of mucosal immune homeostasis [2]. Impairment of the microbial barrier may allow enteric bacteria to cause sepsis [11]. Intestinal microbiota dysbiosis is often seen in patients with sepsis, suggesting its possible contribution in the initiation and/or perpetuation of the disease [12,13]. Regulation of gut microbiota is a delicate balancing act. Given the intestinal dysbiosis and its prominent role in the development of sepsis, improved clinical outcomes may be achieved with FMT in patients with sepsis. However, experience with this procedure in sepsis remains limited. The efficacy of FMT in recurrent CDI encouraged us to investigate the therapeutic value of the strategy in patients with sepsis and the underlying mechanisms. In this article, we describe a case of a patient who developed septic shock and severe diarrhea following vagotomy and report our findings regarding FMT. We also sought to investigate the changes in the identity and abundance of the bacteria in gut microbial communities and to assess relationships between these assemblages and immunologic signatures of the sepsis patient.

## Material and methods

### Ethical approval

The procedure we performed was approved by the Administrative Panel for Medical Research on Human Subjects of Jinling Hospital (the ethics committee of our hospital). The patient gave us her written informed consent to undergo the procedure and to have her case published.

### Case presentation

**Figure 1:**
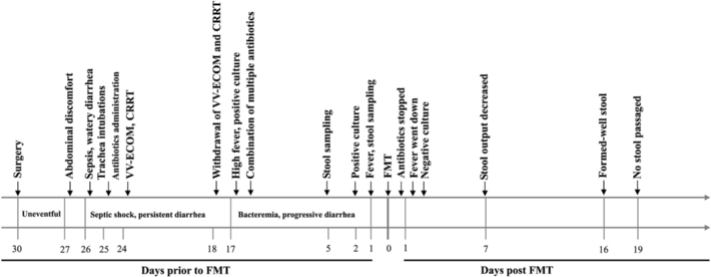
**Treatment timeline of the patient.** The timeline shows the major clinical events during the course of the patient’s treatment. CRRT, Continuous renal replacement therapy; VV-ECMO, Venovenous extracorporeal membrane oxygenation; FMT, Fecal microbiota transplantation.

Our patient was a 44-year-old woman who underwent proximal gastrectomy and bilateral truncal vagotomy for a gastric neuroendocrine tumor. Her immediate postoperative course was uneventful, and no surgical complications, such as anastomotic fistula, were observed. On the third postoperative day, she presented with abdominal discomfort and bloating, nausea and vomiting (Figure 1). On the fourth postoperative day, the patient developed severe, watery diarrhea. Her blood pressure suddenly decreased to 60/38 mmHg for unknown reason. An examination revealed that her body temperature was 37.2°C, her pulse was increased at 126 beats/minute, her respiratory rate was 28 breaths/minute and her blood oxygen saturation was 86%. A complete blood count was obtained, and the results revealed a white blood cell count of 2.9 × 10^9^/L with 83% neutrophils and 13% lymphocytes. Analysis of blood gas showed that the patient’s blood lactate level was 8.2 mmol/L and base excess was −9.2. Serum level of C-reactive protein (CRP) was 143.4 mg/L. Blood and urine were collected for bacterial culture, and both test results were negative. The patient was urgently transferred to the ICU. On the fifth postoperative day, her temperature rose to 39.6°C and her pulse rate was 145 beats/minute, and she developed respiratory distress (Additional file 1). Endotracheal intubation was performed to allow mechanical ventilation (Figure 1). Her degree of diarrhea was aggravated, with a passage of greenish, watery liquid exceeding 1,800 ml. Her white blood cell count had increased to 7.9 × 10^9^/L, and the proportion of neutrophils had risen to 98%. Her platelet count had decreased, and a coagulation test showed a prolonged time to coagulation. Sulbactam, ornidazole, vecuronium, norepinephrine, hydrocortisone and intensive intravenous fluids were administered, owing to concern about septic shock [14]. The patient was then treated with venovenous extracorporeal membrane oxygenation (VV-ECMO) via percutaneous access and continuous renal replacement therapy (CRRT). During the following days, the patient’s vital signs and clinical condition gradually stabilized. VV-ECMO and CRRT were stopped on the 12th postoperative day, but the patient’s diarrhea did not shown any improvement. On the next day, her fever reoccurred, with a peak temperature of 38.7°C (Additional file 1). Her white blood cell count rose further to 18.0 × 10^9^/L with 89% neutrophils and 10% lymphocytes. Her blood cultures now yielded *Acinetobacter baumannii* and *Enterococcus faecalis*, revealing a polymicrobial sepsis. Multiple antibiotics, including imipenem, caspofungin, penicillin and/or linezolid, and even probiotic bifidobacteria, were administered. Abdominal computed tomography (CT) was performed on days 15 and 22 postsurgery, and no intraabdominal free fluid or abscesses was noted (Additional file 2 and Additional file 3). The therapeutic strategies with antibiotics and probiotic were continued until 28 days, when the patient still had a fever (38.3°C) and the volume of watery stools reached 2,855 ml. On that day, the patient’s blood culture grew *Propionibacterium acnes*. Her white blood cell count returned to normal, but her CRP level remained as high as 103.3 mg/L. The timeline shown in Figure 1 depicts the temporal course of our patient’s treatment.

### Analysis of fecal microbiota

Considering the possibility of intestinal dysbiosis, we applied 16S rRNA gene-based molecular techniques to characterize the fecal bacterial composition in our patient according to a procedure described previously [15]. The partial fragments of the 16S rRNA gene were amplified using universal primers targeting the hypervariable V3 region and were separated by denaturing gradient gel electrophoresis (DGGE) [15]. The predominant bands were sequenced to gain the closest bacterial relatives using the Basic Local Alignment Search Tool, and they were phylogenetically analyzed with *MEGA* 4.0 software [16,17].

### Fecal microbiota transplantation

**Figure 2:**
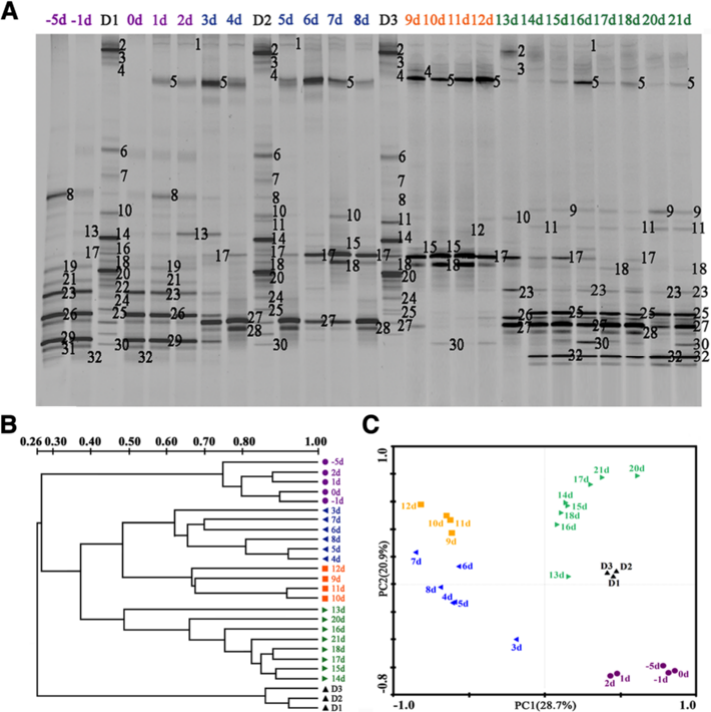
**Molecular assessments of the fecal bacterial microbiota in the patient. (A)** Representative fingerprints of denaturing gradient gel electrophoresis (DGGE) gels for fecal microbiota. The figures 1 to 32 represent the predominant bands for DNA sequencing. The closest bacterial phylotypes corresponding to these bands are shown in Additional file 5. **(B)** Dendrogram generated from our DGGE analysis. The clustering profiles were obtained from DGGE analysis using the unweighted pair group method with arithmetic average. The metric scale indicates the degree of similarity (%). D1 to D3 denote the fecal specimens from the donor. −5d, −1d and 0d to 21d represent the fecal samples collected from the patient 5 and 1 days before and 0 to 21 days after fecal microbiota transplantation, respectively. The 19d data are missing because there was no fecal output at 19 days after infusion of donor feces. **(C)** Plot generated from the relative abundance of DGGE bands depicting our principal component analysis of the patient’s fecal microbiota.

On the basis of the molecular analysis, the patient’s microbiota was extensively perturbed (Figure 2), raising an interesting possibility of using FMT in the management of her disorder. Therefore, the infusion of donor feces was performed in the patient. The patient’s brother (40 years old) was selected as the donor of fecal microbiota. He was screened to exclude the risks for blood-borne communicable diseases. His fecal samples were validated as negative for common stool pathogens. The feces (70 g) freshly collected on the day of infusion were diluted with sterile saline (350 ml). The homogenized solution was filtered twice through a presterilized metal sieve. The filtrates (120 ml) were infused into the patient via a nasogastric tube on the 30th day after her surgery. The patient’s stool was daily collected, and an aliquot (220 mg) of each sample was immediately stored at −70°C until DNA extraction. Her fecal microbiota was assessed using the protocol stated above.

### Flow cytometric analysis

Blood specimens were collected from the patient and from healthy subjects. After activation with phorbol myristate acetate and ionomycin, immunostaining was performed using fluorescein-labeled monoclonal antibodies against CD4, interferon γ (INF-γ), interleukin (IL)-4 and IL-17 (BD Biosciences, San Jose, CA, USA) [18]. Cells were run on a BD FACSCalibur flow cytometer, and the data were analyzed with CellQuest software (BD Biosciences). A logical gate combining CD4^+^ cells and their scatter properties was used for the phenotypes of T helper type 2 (Th2) and Th17 cells.

### Assay of serum cytokines

Serum concentrations of tumor necrosis factor α (TNF-α), IL-1β, IL-6, IL-10, IL-18, IFN-γ and high-mobility group box 1 (HMGB1) protein were measured using enzyme-linked immunosorbent assay kits (R&D Systems, Abingdon, UK).

### Statistical analysis

The relative intensity of each band was expressed as a proportion (%) of the sum of all fragments in the same lane of the gel [15]. Principal component analysis (PCA) was conducted with CANOCO software for Windows 4.5 (Microcomputer Power, Ithaca, NY, USA). Correlation between two variances was estimated using linear regression analysis with a Pearson test. A *P-*value <0.05 was considered significant.

## Results

### Intestinal microbiota dysbiosis

**Figure 3:**
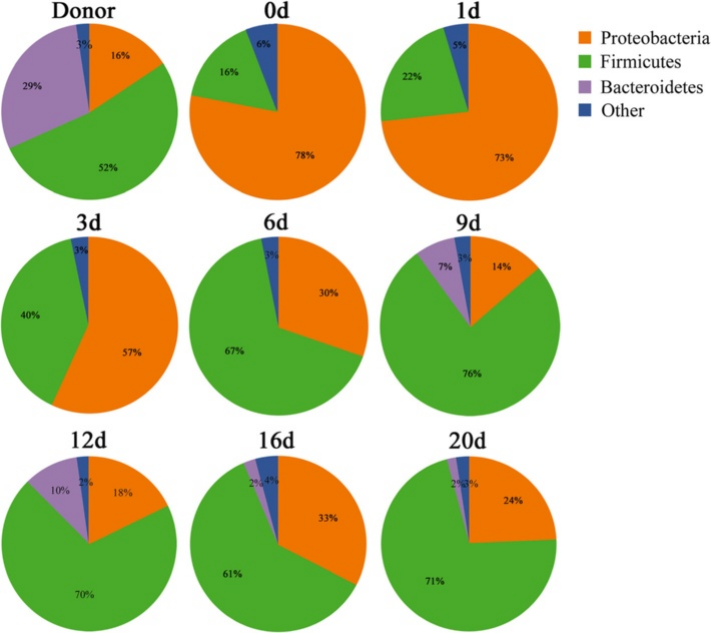
**Determination of the predominant bacterial composition in the fecal microbiota at the phylum level.** Representative time points were selected to show the variations of the microbiota composition following fecal microbiota transplantation.

**Figure 4:**
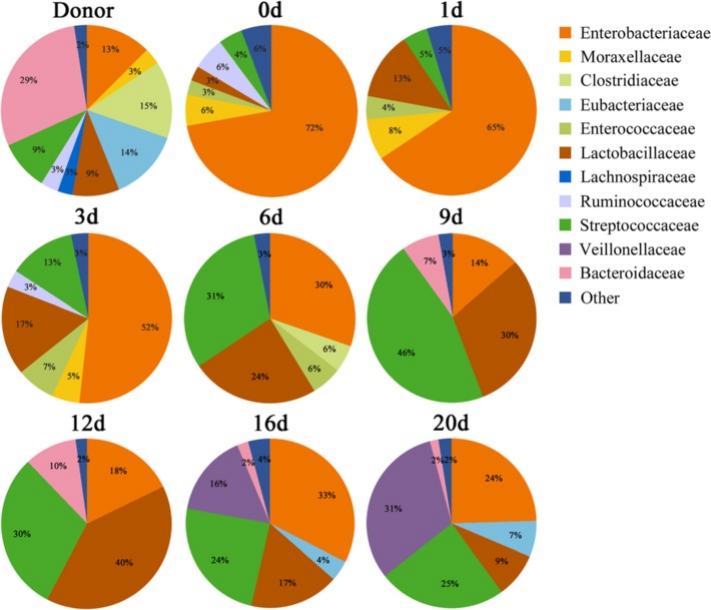
**Changes of the predominant bacterial composition at the family level after fecal microbiota transplantation.**

**Figure 5:**
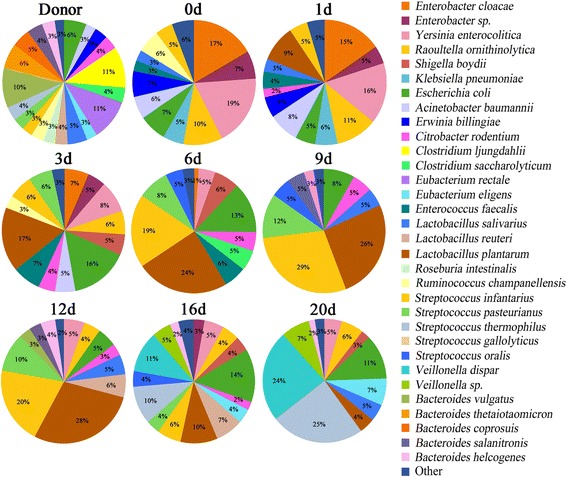
**Variations of the major bacterial species in the fecal microbiota after fecal microbiota transplantation.**

As shown in the molecular fingerprinting (Figure 2A,B), the patient’s microbiota was extensively disturbed, with a very low similarity compared to that of the healthy subject. PCA provided clearer evidence for this dysbiosis (Figure 2C). The observations were further confirmed by compositional analysis of the predominant bacterial taxa. The most striking changes in the microbiota were the depletion in the phyla Firmicutes (16% vs. 52%) and Bacteroidetes (0% vs. 29%) and a dramatic expansion in the phylum Proteobacteria (78% vs. 16%) in comparison to those of the healthy control (Figure 3). The reduction in Firmicutes was due mainly to significant depletion of the families Eubacteriaceae and Clostridiaceae in particular, as well as of the families Ruminococcaceae and Lachnospiraceae (Figure 4). The phylum Bacteroidetes was not detected in the patient’s microbiota, which was attributable mainly to absence of the family Bacteroidaceae. Altered Proteobacteria was featured by expansion of the Enterobacteriaceae family members with proinflammatory potential, including *Enterobacter cloacae* (band 29), *Yersinia enterocolitica* (band 26), *Raoultella ornithinolytica* (band 23), *Klebsiella pneumoniae* (band 19), *Enterobacter* sp. (band 31) and *Acinetobacter baumannii* (band 8) (Figure 5, Additional file 4 and Additional file 5).

### Improvement of clinical outcomes

**Figure 6:**
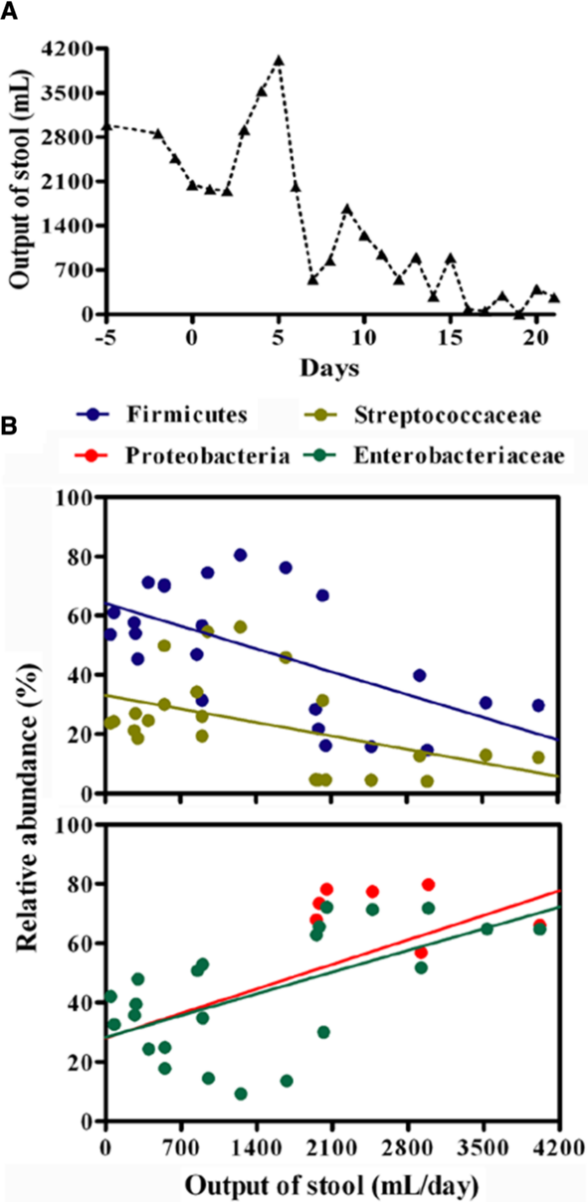
**Stool output of the patient and the association with specific bacterial phylogroups. (A)** Variations of fecal output in the patient after fecal microbiota transplantation. Total volume of the stool was documented each day. **(B)** Correlations of the relative proportions of Firmicutes, Streptococcaceae, Proteobacteria and Enterobacteriaceae with stool output per day. Correlation between two variances was estimated using linear regression analysis with a Pearson test.

On the basis of the findings described above, we applied FMT for the treatment of sepsis and severe diarrhea in our patient when conventional strategies with antibiotics and probiotics failed. The septic symptoms and diarrhea were expected to improve after FMT (Additional file 1). Our patient’s body temperature to 37.1°C at 1 day from 37.8°C prior to the infusion. There was no recurrence of septic symptoms in the following days. Cultures of the patient’s blood became sterile. Her stool output declined after 7 days (Figure 6A). Her stools became well formed, and the frequency and volume of her stool returned to normal after 16 days. Our data provide evidence for the beneficial effects of FMT on sepsis and diarrhea in our patient.

### Reshaping of the gut bacterial consortia by fecal microbiota transplantation

In an effort to clarify the possible mechanism underlying the clinical benefits achieved in our patient, we evaluated the temporal changes of the bacterial microbiota following FMT. Analysis of DGGE banding patterns showed that the structure of the microbiota, especially 3 days later, was dramatically altered, as revealed by a phylogenetic cluster separated from those before the infusion of donor feces (Figure 2A,B). After 14 days, the similarities between the microbiota rose to more than 66.4% (Figure 2B), suggesting that the community structures trended toward stability. The data obtained from PCA further support these results (Figure 2C).

We next identified the variations of our patient’s microbiota composition following FMT. As compared with day 0, a profound expansion in Firmicutes and a striking reduction in Proteobacteria were noted in the microbiota, especially 6 days post-FMT (Figure 3). The families Streptococcaceae, Lactobacillaceae, Eubacteriaceae and Veillonellaceae were significantly increased, contributing mostly to the recovery of the phylum Firmicutes (Figure 4). *Streptococcus pasteurianus* (band 18), a main component of the family Streptococcaceae, was presented at 2 days postinfusion and persisted in a higher proportion until 12 days (Figure 5). In addition, the commensal organisms, including *Streptococcus thermophilus* (band 25), *Lactobacillus plantarum* (band 5), *Eubacterium eligens* (band 11) and *Veillonella dispar* (band 32), colonized and dominated in the microbiota after 14 days. In contrast to this, the pathobionts of the family Enterobacteriaceae, especially *Enterobacter cloacae*, *Yersinia enterocolitica*, *Raoultella ornithinolytica*, *Klebsiella pneumoniae* and *Enterobacter* sp., were significantly depleted. *Citrobacter rodentium* (band 1), which was absent before FMT, was introduced after the treatment. The temporal shifts in the composition of microbial communities were also seen in the phylogenetic analysis (Additional file 6). Together, the remarkable modification and reestablishment of the intestinal microbiota in the patient may have been primarily attributable to donor feces infusion.

### Immunomodulatory effect of therapeutic microbial manipulation

**Figure 7:**
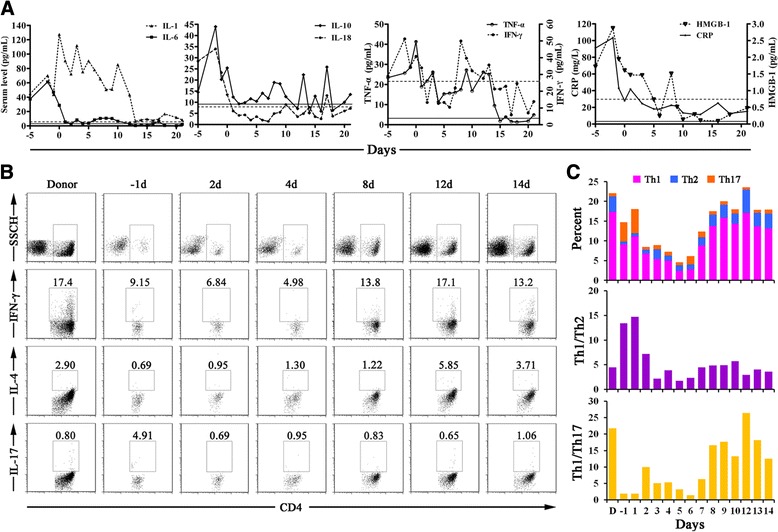
**Serum levels of inflammatory mediators in the patient before and after fecal transplantation. (A)** Graphs depict the serum levels of inflammatory mediators in the patient before and after fecal transplantation. All data are expressed as means of duplicates. The solid and dotted lines represent the mean values of serum concentrations in healthy subjects. **(B)** Representative histograms illustrate flow cytometry results as percentage of CD4^+^ T cells producing interferon γ (side scatter height (SSCH); IFN-γ; T helper type 1 (Th1)), interleukin (IL)-4 (Th2) and IL-17 (Th17). **(C)** Changes in T helper cell subpopulations in the patient before and after fecal transplantation are depicted.

Before fecal transplantation, the patient showed an uncontrolled systemic inflammatory response, as revealed by dramatic elevation in serum inflammatory mediators (Figure 7A). Serum levels of TNF-α, IL-6 and IL-1β were significantly increased, by 7.5-, 18.1- and 10.4-fold of the upper limits of normal, respectively. Also, the patient’s IFN-γ, IL-10, IL-18 and HMGB1 values were markedly elevated. After infusion of donor feces, there was a marked decrease in IL-6, IL-10 and IFN-γ, especially after 12 days. Appreciable reductions in TNF-α and IL-1β were seen after 13 days. The patient’s serum CRP declined significantly after 10 days. The differentiation of Th lymphocytes was strikingly influenced by FMT (Figure 7B). The proportions of Th1 and Th2 cells were restored to baseline levels after 8 days, and their ratio went back simultaneously. Th17 cells were reduced on day 2, and the Th1/Th17 ratio returned to normal 8 days later.

### Gut bacterial phylogroups correlate with diarrhea and inflammatory markers

**Figure 8:**
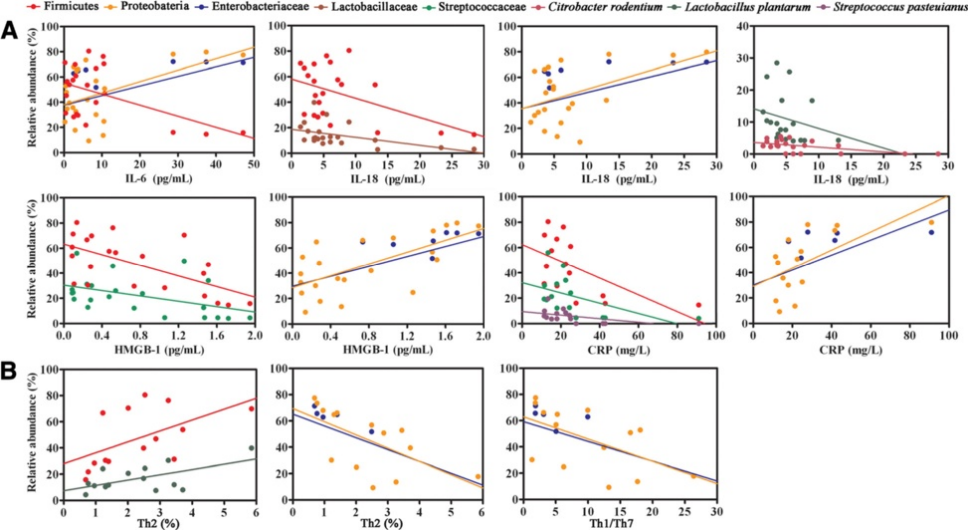
**Correlation analyses between specific bacterial phylogroups and immunological parameters.** Graphs depicting the taxon abundance of many members within the phyla Firmicutes and Proteobacteria, which had strong associations with inflammatory markers **(A)** and the proportions of circulating Th cells **(B)**. CRP, C-reactive protein; HMGB-1, High-mobility group box 1; IL, Interleukin; IFN, interferon; Th, T helper cell.

We next estimated the links between the bacterial taxa and clinical signatures. The taxon abundance of Firmicutes and Streptococcaceae exhibited negative correlations with the output of stools, whereas the taxa Proteobacteria and Enterobacteriaceae positively correlated with it (Figure 6B). Strong associations between the relative abundance of bacterial taxa and inflammatory markers were also found (Figure 8A). The phylum Firmicutes and the families Streptococcaceae and Lactobacillaceae were negatively correlated with many inflammatory markers of sepsis progression, such as IL-6, IL-18, HMGB1 and CRP. Moreover, some members of the phylum Firmicutes also correlated positively with these parameters. Conversely, the taxon abundance of Proteobacteria and Enterobacteriaceae was positively correlated with the parameters. Additionally, some specific bacterial taxa showed close associations with the changes in the distribution of Th cell subsets (Figure 8B). The phylum Firmicutes and the species *Lactobacillus plantarum* were positively associated with the proportion of Th2 cells, whereas Proteobacteria and Enterobacteriaceae negatively correlated with it. Proteobacteria and Enterobacteriaceae were negatively correlative with the Th1/Th17 ratio.

## Discussion

In this article, we describe a case of a patient who had episodes of sepsis and severe diarrhea following vagotomy, and we report the patient’s clinical outcomes after treatment with FMT. Our patient had an uncontrollable inflammatory response and sepsis after undergoing a vagotomy. Dysbiosis of intestinal microbiota was also seen, prompting us to incorporate FMT into the treatment strategy. Dramatically, our patient’s septic symptoms and severe diarrhea improved after FMT. A significant modification in intestinal microbiota following FMT was observed to be closely associated with her clinical improvement. We also confirmed specific bacterial taxa contributing to this beneficial effect. Together, our data provide initial evidence indicating the efficacy of FMT in the treatment of sepsis and diarrhea and provide novel insights into understanding the basic mechanisms underlying the clinical benefits achieved.

Dysbiosis of intestinal microbiota is commonly seen in patients with sepsis, and it is likely relevant to subsequent clinical sequelae [12,13]. In order to search for potential new therapeutic strategies, we evaluated the structure and composition of the fecal microbiota in our patient. We show that the structure of her gut microbiota was largely disrupted and replaced by an “unfavorable” microbiota with a predominance of opportunistic organisms and deficiency of native residents. The majority of the dysbiotic microbiota was dominated by pathobionts of Proteobacteria and, more especially, of Enterobacteriaceae, accounting for more than three-fourths of the whole community, which is far higher than the presentation of the healthy subjects [19,20]. In contrast, Firmicutes and Bacteroidetes, the two major taxa of a normal bacterial community, were greatly reduced in our patient. Most Eubacteriaceae, Ruminococcaceae and Lachnospiraceae within Firmicutes are beneficial to intestinal epithelial integrity [21,22]. Apparently, the dysbiosis of gut microbiota is due to disturbance of the whole bacterial population rather than to overgrowth of several pathogenic organisms. It may act synergistically with the systemic inflammatory response to participate in the propagation of sepsis. These findings enabled us to better understand the role of the intestinal microbial barrier in sepsis and also guided us to treat the patient with microbiota-directed approaches.

Sepsis and septic shock are life-threatening complications attributed to the host immune system response against microbial pathogens and/or their products, such as endotoxin [23]. Among patients with sepsis who have a positive bacterial culture, the Gram-negative bacteria account for about 60% of cases, most of which originate from the gut [11,24]. An increasing body of evidence supports the hypothesis of gut-derived organisms as a major source of sepsis, especially when no other apparent infectious focus can be identified [11,24-26]. Leaky gut following abdominal surgical intervention may cause translocation of enteric microorganisms into the circulation, further triggering the systemic inflammatory response and subsequent lethal complications [27,28]. Our patient developed septic shock 4 days after undergoing surgery, and no anastomotic fistula was present. In addition, no other infectious focus, such as intraabdominal free fluid or abscesses, was seen by abdominal CT scanning (Additional file 2 and Additional file 3). The patient’s blood cultures grew enteric organisms, including *Acinetobacter baumannii*, *Enterococcus faecalis* and *Propionibacterium acnes*. On the basis of the data, our patient presumably had gut-derived sepsis due to intestinal bacteria, likely as a result of the loss of the intestinal barrier after surgery.

FMT represents the therapeutic protocol that allows the reconstitution of a disrupted intestinal microbial community [7,29]. The perturbation of gut microbiota is probably relevant for the etiology of sepsis, making FMT an attractive therapeutic strategy for patients with sepsis. However, the availability of FMT in sepsis and the mechanisms underlying its effects remain largely unexplored. In this case report, we describe the use of FMT in a patient with severe sepsis and diarrhea following vagotomy. Remarkably, her septic symptoms improved, and her severe diarrhea was effectively controlled. More important is the dramatic relief of systemic inflammation and reestablishment of innate immune equilibrium. To understand the mechanism of the procedure in more detail, we characterized the gut microbiota of the patient after the FMT intervention, and we evaluated associations between specific bacterial taxa and clinical outcomes. Following the treatment, the patient’s bacterial population shifted strikingly toward a normal commensal pattern, supporting the growth of the commensal Firmicutes while eliminating a subset of Proteobacteria. The resident bacteria derived from the donor, such as *Streptococcus pasteurianus*, *Streptococcus thermophilus*, *Lactobacillus plantarum* and *Eubacterium eligens*, dominated in the reconstituted microbiota. Equally crucial is the significant depletion of proinflammatory pathobionts in Proteobacteria, including *Enterobacter cloacae* and other *Enterobacter* spp., *Yersinia enterocolitica*, *Raoultella ornithinolytica* and *Klebsiella pneumoniae.* The data suggest that FMT may induce a significant modification in intestinal microbiota and facilitate reshaping a microecological defense barrier in the patient. Further analysis showed that selective shifts of some specific bacteria following FMT are associated with clinical improvements. Several members within the Firmicutes are negatively associated with inflammatory markers, whereas the pathobionts of Proteobacteria exhibit positive correlations with them. Furthermore, the phyla Firmicutes and Proteobacteria are associated with proportions of Th cells. These findings indicate that elevated abundance of multiple, diverse microbial taxa within the Firmicutes and apparent depletion of Proteobacteria following FMT are significantly linked to alleviation of the inflammatory response and recovery of innate immune homeostasis. Eradication of an inappropriate immune response parallels the development of the new microbial equilibrium, providing compelling evidence for a causative role of FMT. Our data demonstrate that FMT not only repairs an enteric microbial barrier but also feeds back to modulate host immunity, resulting in improvement of clinical outcomes.

Recently, the vagal innervation of the gastrointestinal tract has been proposed to be involved in the regulation of the inflammatory response in animal models of sepsis [30,31]. Vagotomies are frequently performed in patients with gastrectomy. To date, no data are available supporting the effect of vagotomy on the development of sepsis in clinical practice. Our patient experienced episodes of septic shock 4 days after the surgical intervention; however, it was not well proven that the pathogenesis of the sepsis was causally related to the vagotomy.

## Conclusions

In this article, we report what is, to the best of our knowledge, the first case in the literature of a patient with sepsis who underwent vagotomy and subsequently received FMT, and we present preliminary results. The findings presented here imply that FMT may be an effective means of treating patients with severe sepsis and diarrhea. We demonstrate that targeted manipulation of intestinal microbiota through FMT may elicit microecological and immunological mechanisms to maintain intestinal homeostasis and protect the patient. This report provides a wonderful example of a proof-of-concept study indicating that FMT may represent an ideal therapeutic alternative to control systemic inflammation in patients with sepsis. Despite the favorable outcome, our findings were obtained from a single patient and therefore may be considered preliminary. It may be difficult to prove the causal relationship between the recovery of intestinal microbial homeostasis and clinical benefits in our patient. Further investigation with randomized clinical trials is warranted to validate the efficacy of this procedure for sepsis and for broader clinical use.

## Key messages

- Septic symptoms and severe diarrhea in our patient were resolved following infusion of donor feces.
- The patient’s clinical improvements were due at least in part to recovery of intestinal microbial homeostasis by FMT.

## Consent

Written informed consent was obtained from the patient for publication of this manuscript and any accompanying images. A copy of the written consent is available for review by the Editor-in-Chief of this journal.

## Additional files

Additional file 1:
**Clinical characteristics and laboratory parameters of the patient.**
Additional file 2:
**Computed tomographic scans of the abdomen.** Representative images are selected to show no intraabdominal free fluid or abscesses. Additional file 3:
**Images of the patient’s abdomen obtained by computed tomographic scanning.** Detailed cross-sectional images of the abdomen obtained before and after the vagotomy show no intraabdominal free fluid or abscesses. Additional file 4:
**Phylogenetic tree generated from the partial 16S rDNA sequences obtained from fecal microbiota.** The sequences were aligned with closely related 16S rDNA sequences retrieved from the GenBank database using the Basic Local Alignment Search Tool (BLAST). The tree was constructed by using the sequences of the predominant species in the feces with MEGA software. The scale bar represents the genetic distance. Additional file 5:
**The sequence analysis of DGGE bands from the feces.**
Additional file 6:
**Variations in phylogenetic distribution of 16S rDNA sequences following fecal infusion.** The diagrammatic phylogenetic tree presents a summary of the rRNA sequences obtained from DGGE bands in this study. Phyla are named to the left of the tree, and lower taxonomic levels are given to the right. The number in the clade is designated as the relative proportion (%) in the whole fecal microbiota.

## Abbreviations

CDI: *Clostridium difficile* infection
CRP: C-reactive protein
CRRT: Continuous renal replacement therapy
CT: Computed tomography
DGGE: Denaturing gradient gel electrophoresis
FMT: Fecal microbiota transplantation
HMGB1: High-mobility group box 1
ICU: Intensive care unit
IFN-γ: Interferon γ
IL: Interleukin
PCA: Principal component analysis
Th: T helper
TNF-α: Tumor necrosis factor α
VV-ECMO: Venovenous extracorporeal membrane oxygenation
