# Supplementary material for: Successful treatment of severe sepsis and diarrhea after vagotomy utilizing fecal microbiota transplantation: a case report
Source: Crit Care. 2015 Feb 9;19(1):37. doi: 10.1186/s13054-015-0738-7 (PMC4346118; doi:10.1186/s13054-015-0738-7)
Supplement: Additional file 1: — Clinical characteristics and laboratory parameters of the patient. [file 13054_2015_738_MOESM1_ESM.pdf]

Additional file 1 Clinical characteristic and laboratory parameters of the patient

**Day of gastrectomy**	**4**	**5**	**13**	**29**	**30**	**31**	**32**	**33**	**34**	**35**	**36**	**37**
**Day of fecal transplantation**	**-26**	**–25**	**–17**	**–1**	**0**	**1**	**2**	**3**	**4**	**5**	**6**	**7**
Temperature (℃)	36.3-37.2	37.8-39.6	37.2-38.7	36.5-38.0	36.5-37.8	36.6-37.1	36.5-37.3	36.4-36.9	36.0-36.6	36.0-36.3	36.5-37.2	36.5-37.1
Heart rate (bpm)	96-126	89-145	86-118	73-91	76-112	74-92	76-89	79-88	76-89	76-86	81-100	76-93
Respiratory rate (beats/min)	17-28	15-27	12-21	15-24	15-30	14-26	15-33	18-22	16-24	16-25	14-26	16-23
Blood pressure (mmHg)	60-122/ 38-64	77-156/ 52-80	118-160/ 76-90	102-124/ 54-71	97-125/ 55-68	90-123/ 50-75	94-125 /47-69	90-113/ 54-67	93-119/ 54-75	95-112/ 53-72	93-114/ 56-73	96-125/ 56-71
Blood oxygen saturation (%)	86-100	87-100	94-100	98-100	96-100	96-100	97-100	94-100	97-100	97-100	98-100	98-100
pH	7.366	7.348	7.473	7.367	7.344	7.430	7.360	7.373	7.399	7.369	7.298	7.395
PaO_2 (mmHg)	67.4	83.3	57.0	77.2	118.0	147.0	116.0	93.7	138.0	91.7	142.0	110.0
$PaCO_2$ (mmHg)	26.2	29.6	36.2	31.8	30.3	30.0	32.8	26.8	28.2	29.0	23.1	26.0
BE	-9.2	-8.3	-7.2	-6.2	-8.2	-4.0	-6.2	-8.6	-6.3	-7.6	-4.1	-7.9
Lac (mmol/L)	8.2	8.3	5.1	3.7	3.4	1.1	1.2	0.9	0.9	1.2	1.1	1.3
C-reactive protein (mg/L)	143.4	55.0	64.6	42.8	27.8	41.7	n.d.	24.4	n.d.	18.0	17.9	n.d.
Daily stool volume (mL)	700	1800	2350	2470	2050	1980	1955	2920	3530	4020	2020	550
**Hematological analysis**												
White blood cell count ($\times 10^9$/L)	2.9	7.9	18.0	6.8	9.4	6.6	6.5	7.7	8.7	6.9	6.4	6.5
Neutrophil percentage (%)	83	98	89	58	64	71	60	64	66	61	55	57
Lymphocyte percentage (%)	13	2	10	30	26	20	32	29	29	32	37	36

Platelet ($\times 10^9$/L)	138	62	76	280	281	273	281	272	294	299	309	312
**Liver function**												
Total protein (g/L)	65.5	45.8	60.4	53.8	52.2	50.5	50.5	48.1	49.8	50.8	52.9	55.1
Albumin (g/L)	34.2	25.6	31.1	34.7	35.4	35.8	35.8	34.1	34.0	33.4	36.4	34.5
Total bilirubin (μmol/L)	8.5	17.6	42.0	24.2	18.7	16.2	12.4	11.9	12.1	9.3	11.0	9.9
Direct bilirubin (μmol/L)	0	15.5	29.6	9.9	6.8	6.3	3.5	4.3	4.5	2.2	3.7	0.7
Indirect bilirubin (μmol/L)	8.5	2.1	12.4	14.4	12.0	9.9	8.9	7.6	6.7	7.1	7.3	9.2
**Renal function**												
Creatinine (μmol/L)	94	131	51	37	34	36	38	34	32	32	35	29
Urea N (mmol/L)	4.6	5.3	7.3	5.6	5.4	4.6	6.2	6.4	5.9	7.0	5.7	7.0
Uric acid (μmol/L)	259	249	54	146	126	140	112	116	114	109	143	100
**Blood coagulation test**												
Prothrombin time (s)	17.6	24.5	14.1	13.9	n.d.	13.4	13.7	14.4	15.3	n.d.	14.0	14.2
Partial thromboplastin time (s)	48.2	>120.0	29.0	30.9	n.d.	32.1	34.6	33.6	37.2	n.d.	31.6	28.9
International normalized ratio	1.52	2.11	1.22	1.21	n.d.	1.16	1.19	1.25	1.33	n.d.	1.21	1.23
Fibrinogen (mg/dL)	226	171	145	289	n.d.	234	265	279	339	n.d.	306	286

(Continued)

**Day of gastrectomy**	**38**	**39**	**40**	**41**	**42**	**43**	**44**	**45**	**46**	**47**	**48**	**50**	**51**
**Day of fecal transplantation**	**8**	**9**	**10**	**11**	**12**	**13**	**14**	**15**	**16**	**17**	**18**	**20**	**21**
Temperature (℃)	36.0-37.0	36.5	36.3-37.6	36.0-37.4	36.7-37.3	36.0-37.2	36.7-37.0	36.1-37.1	36.0-36.8	36.5-37.2	36.3-36.7	36.0-36.5	36.0-36.8
Heart rate (bpm)	75-87	70-83	63-85	71-88	69-90	71-86	73-84	72-92	79-92	72-91	70-94	60-83	67-82
Respiratory rate (beats/min)	14-22	13-23	16-26	13-29	13-24	16-26	14-24	16-23	15-28	17-26	14-24	12-23	16-27
Blood pressure (mmHg)	93-114/ 54-68	99-111/ 52-79	97-111/ 59-75	101-117/ 63-78	102-120/ 62-77	98-121/ 63-78	94-114/ 58-79	92-116/ 59-79	92-113/ 58-78	95-124/ 61-80	95-125/ 60-88	93-123/ 62-84	97-121/ 63-85
Blood oxygen saturation (%)	96-100	97-100	98-100	98-100	93-100	98-100	100	94-100	98-100	99-100	95-100	97-100	96-100
pH	7.384	7.392	7.323	7.298	7.416	7.465	7.447	7.508	7.468	7.479	7.483	n.d	7.453
PaO_2 (mmHg)	116.0	126.0	118.0	134.0	75.9	89.4	122.0	73.5	89.1	54.4	147.0	n.d	134.0
$PaCO_2$ (mmHg)	31.9	27.0	30.1	26.5	36.3	31.2	33.5	32.5	31.7	33.4	34.6	n.d	37.0
BE	-5.3	-10.8	-9.5	-12.4	-0.9	-0.8	-0.4	2.7	-0.6	1.2	2.4	n.d	1.9
Lac (mmol/L)	0.7	0.5	0.5	0.7	1.5	1.0	0.6	0.9	1.0	1.1	0.7	n.d	1.0
C-reactive protein (mg/L)	22.50	21.30	13.39	16.80	11.60	11.43	n.d	n.d	25.00	n.d	12.62	n.d	15.00
Daily stool volume (mL)	850	1675	1250	950	550	900	280	900	80	50	300	400	270
**Hematological analysis**													
White blood cell count ($\times 10^9$/L)	5.3	4.8	6.1	5.3	4.5	4.1	4.9	4.5	3.5	4.2	4.0	n.d	2.7
Neutrophil percentage (%)	53	45	46	35	42	35	45	43	30	30	20	n.d	n.d
Lymphocyte percentage (%)	40	45	41	54	46	56	46	49	59	61	68	n.d	69
Platelet ($\times 10^9$/L)	282	295	354	334	295	252	258	134	161	185	156	n.d	160

**Liver function**													
Total protein (g/L)	48.30	51.20	54.80	59.40	53.50	52.40	51.00	53.14	54.10	55.51	55.88	n.d	49.90
Albumin (g/L)	32.50	35.40	35.60	31.80	35.10	34.10	33.60	34.05	34.60	35.33	34.65	n.d	31.90
Total bilirubin (μmol/L)	11.80	13.85	12.01	11.80	12.69	14.16	12.38	12.80	12.64	14.38	14.91	n.d	11.69
Direct bilirubin (μmol/L)	1.90	2.04	1.48	7.80	1.35	2.93	1.73	1.25	1.36	2.44	6.02	n.d	4.71
Indirect bilirubin (μmol/L)	9.90	11.81	10.53	4.00	11.34	11.23	10.65	11.55	11.28	11.94	8.89	n.d	6.98
**Renal function**													
Creatinine (μmol/L)	36.00	41.00	43.00	58.00	52.00	48.00	41.00	38.49	39.00	36.08	39.19	n.d	33.00
Urea N (mmol/L)	7.10	4.80	2.50	0.90	1.80	1.10	0.90	0.83	2.10	1.46	1.86	n.d	1.40
Uric acid (μmol/L)	111	168	200	224	248	203	157	137	158	145	174	n.d	147
**Blood coagulation test**													
Prothrombin time (s)	14.7	14.4	13.5	13.6	13.0	13.0	11.6	11.0	12.2	12.1	12.1	n.d	13.0
Partial thromboplastin time (s)	30.1	28.5	25.6	28.2	22.5	28.5	23.8	21.4	25.7	30.5	29.2	n.d	32.6
International normalized ratio	1.28	1.25	1.17	1.18	1.13	1.13	1.01	0.96	1.06	1.05	1.05	n.d	1.13
Fibrinogen (mg/dL)	279	265	276	257	265	215	203	145	63	250	220	n.d	195

Notes: n.d.: no detected.
