# Supplementary material for: Successful treatment of severe sepsis and diarrhea after vagotomy utilizing fecal microbiota transplantation: a case report
Source: Crit Care. 2015 Feb 9;19(1):37. doi: 10.1186/s13054-015-0738-7 (PMC4346118; doi:10.1186/s13054-015-0738-7)

Additional file 2 Computed tomographic scan of the abdomen. Representative images are selected to show intraabdominal free fluid and abscesses.

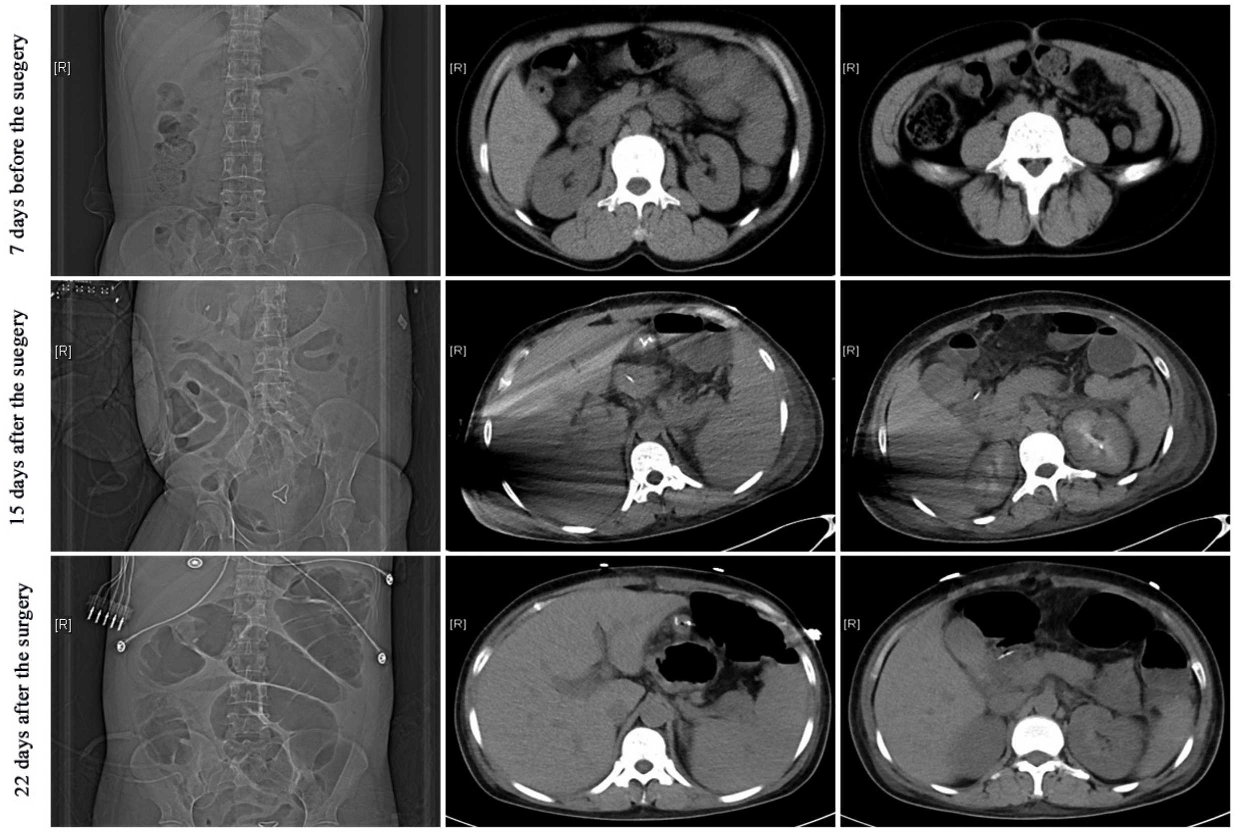

Supplement: Additional file 2: — Computed tomographic scans of the abdomen. Representative images are selected to show no intraabdominal free fluid or abscesses. [file 13054_2015_738_MOESM2_ESM.pdf]
