# Supplementary material for: Successful treatment of severe sepsis and diarrhea after vagotomy utilizing fecal microbiota transplantation: a case report
Source: Crit Care. 2015 Feb 9;19(1):37. doi: 10.1186/s13054-015-0738-7 (PMC4346118; doi:10.1186/s13054-015-0738-7)
Supplement: Additional file 3: — Images of the patient’s abdomen obtained by computed tomographic scanning. Detailed cross-sectional images of the abdomen obtained before and after the vagotomy show no intraabdominal free fluid or abscesses. [file 13054_2015_738_MOESM3_ESM.pdf]

Additional file 3 Phylogenetic tree generated from the partial 16S rDNA sequences obtained from the fecal microbiotas. The sequences were aligned with closely related 16S rDNA sequences retrieved from GenBank database using the Basic Local Alignment Search Tool. The tree was conducted by the sequences of the predominant species in the feces through the *MEGA* software. The scale bar represents the genetic distance.

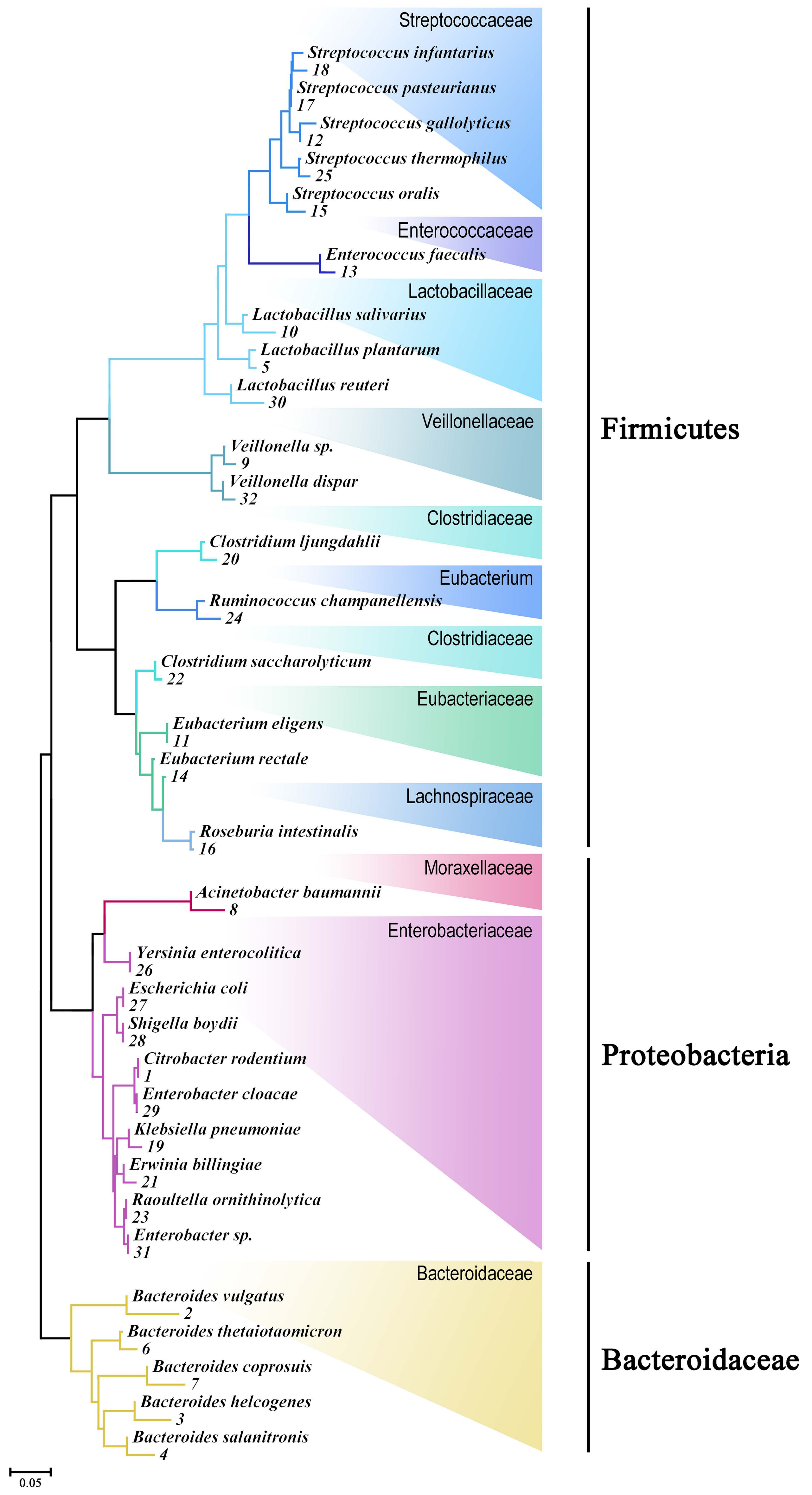
Streptococcaceae
Streptococcus infantarius
18
Streptococcus pasteurianus
17
Streptococcus gallolyticus
12
Streptococcus thermophilus
25
Streptococcus oralis
15
Enterococcaceae
Enterococcus faecalis
13
Lactobacillaceae
Lactobacillus salivarius
10
Lactobacillus plantarum
5
Lactobacillus reuteri
30
Veillonellaceae
Veillonella sp.
9
Veillonella dispar
32
Clostridiaceae
Clostridium ljungdahlii
20
Eubacterium
Ruminococcus champanellensis
24
Clostridiaceae
Clostridium saccharolyticum
22
Eubacteriaceae
Eubacterium eligens
11
Eubacterium rectale
14
Lachnospiraceae
Roseburia intestinalis
16
Moraxellaceae
Acinetobacter baumannii
8
Enterobacteriaceae
Yersinia enterocolitica
26
Escherichia coli
27
Shigella boydii
28
Citrobacter rodentium
1
Enterobacter cloacae
29
Klebsiella pneumoniae
19
Erwinia billingiae
21
Raoultella ornithinolytica
23
Enterobacter sp.
31
Bacteroidaceae
Bacteroides vulgatus
2
Bacteroides thetaiotaomicron
6
Bacteroides coprosuis
7
Bacteroides helcogenes
3
Bacteroides salanitronis
4
Firmicutes
Proteobacteria
Bacteroidaceae
0.05
