# Supplementary material for: Successful treatment of severe sepsis and diarrhea after vagotomy utilizing fecal microbiota transplantation: a case report
Source: Crit Care. 2015 Feb 9;19(1):37. doi: 10.1186/s13054-015-0738-7 (PMC4346118; doi:10.1186/s13054-015-0738-7)
Supplement: Additional file 4: — Phylogenetic tree generated from the partial 16S rDNA sequences obtained from fecal microbiota. The sequences were aligned with closely related 16S rDNA sequences retrieved from the GenBank database using the Basic Local Alignment Search Tool (BLAST). The tree was constructed by using the sequences of the predominant species in the feces with MEGA software. The scale bar represents the genetic distance. [file 13054_2015_738_MOESM4_ESM.pdf]

Additional file 4 The sequence analysis of DGGE bands from the feces

Band	Closest relative	Strain	GenBank accession	Identity (%)
1	*Citrobacter rodentium*	ICC168	NC_013716.1	97
2	*Bacteroides vulgatus*	ATCC 8482	NC_009614.1	99
3	*Bacteroides helcogenes*	P 36-108	NC_014933.1	96
4	*Bacteroides salanitronis*	DSM 18170	NC_015164.1	97
5	*Lactobacillus plantarum*	WCFS1	NC_004567.2	99
6	*Bacteroides thetaiotaomicron*	VPI-5482	NC_004663.1	97
7	*Bacteroides coprosuis*	DSM 18011	NZ_CM001167.1	98
8	*Acinetobacter baumannii*	MDR-TJ	NC_017847.1	97
9	*Veillonella sp.*	F0412	NZ_AENU01000007.1	99
10	*Lactobacillus salivarius*	UCC118	NC_007929.1	96
11	*Eubacterium eligens*	ATCC 27750	NC_012778.1	100
12	*Streptococcus gallolyticus*	UCN34	NC_013798.1	99
13	*Enterococcus faecalis*	OG1RF	NC_017316.1	99
14	*Eubacterium rectale*	ATCC 33656	NC_012781.1	99
15	*Streptococcus oralis*	Uo5	NC_015291.1	98
16	*Roseburia intestinalis*	XB6B4	NC_021012.1	97
17	*Streptococcus infantarius*	CJ18	NC_016826.1	99
18	*Streptococcus pasteurianus*	ATCC 43144	NC_015600.1	99
19	*Klebsiella pneumoniae*	MGH 78578	NC_009648.1	99
20	*Clostridium ljungdahlii*	DSM 13528	NC_014328.1	98
21	*Erwinia billingiae*	Eb661	NC_014306.1	97
22	*Clostridium saccharolyticum*	WM1	NC_014376.1	97
23	*Raoultella ornithinolytica*	B6	NC_021066.1	99
24	*Ruminococcus champanellensis*	18P13	NC_021039.1	96
25	*Streptococcus thermophilus*	CNRZ1066	NC_006449.1	99
26	*Yersinia enterocolitica*	subsp. palearctica 105.5R(r)	NC_015224.1	97
27	*Escherichia coli*	UMN026	NC_011751.1	100
28	*Shigella boydii*	CDC 3083-94	NC_011740.1	100
29	*Enterobacter cloacae*	SCF1	NC_014618.1	99
30	*Lactobacillus reuteri*	DSM 20016	NC_009513.1	97
31	*Enterobacter sp.*	638	NC_009436.1	96
32	*Veillonella dispar*	ATCC 17748	NZ_ACIK02000004.1	99
