# Supplementary material for: Successful treatment of severe sepsis and diarrhea after vagotomy utilizing fecal microbiota transplantation: a case report
Source: Crit Care. 2015 Feb 9;19(1):37. doi: 10.1186/s13054-015-0738-7 (PMC4346118; doi:10.1186/s13054-015-0738-7)
Supplement: Additional file 5: — The sequence analysis of DGGE bands from the feces. [file 13054_2015_738_MOESM5_ESM.pdf]

Additional file 5 Variations in phylogenetic distribution of 16S rDNA sequences following fecal infusion. The diagrammatic phylogenetic tree presents a summary of the rRNA sequences obtained from DGGE bands in this study. Phyla are named to the left of the tree and lower taxonomic levels to the right. The number of taxa in a clade is designated as the relative proportion (%) in the whole fecal microbiota. Donor, -1 day, 1 day, 7 days, 14 days, and 21 days indicate the samples of the donor, 1 day before fecal transplantation, and 1, 7, 14 as well as 21 days after the treatment, respectively.

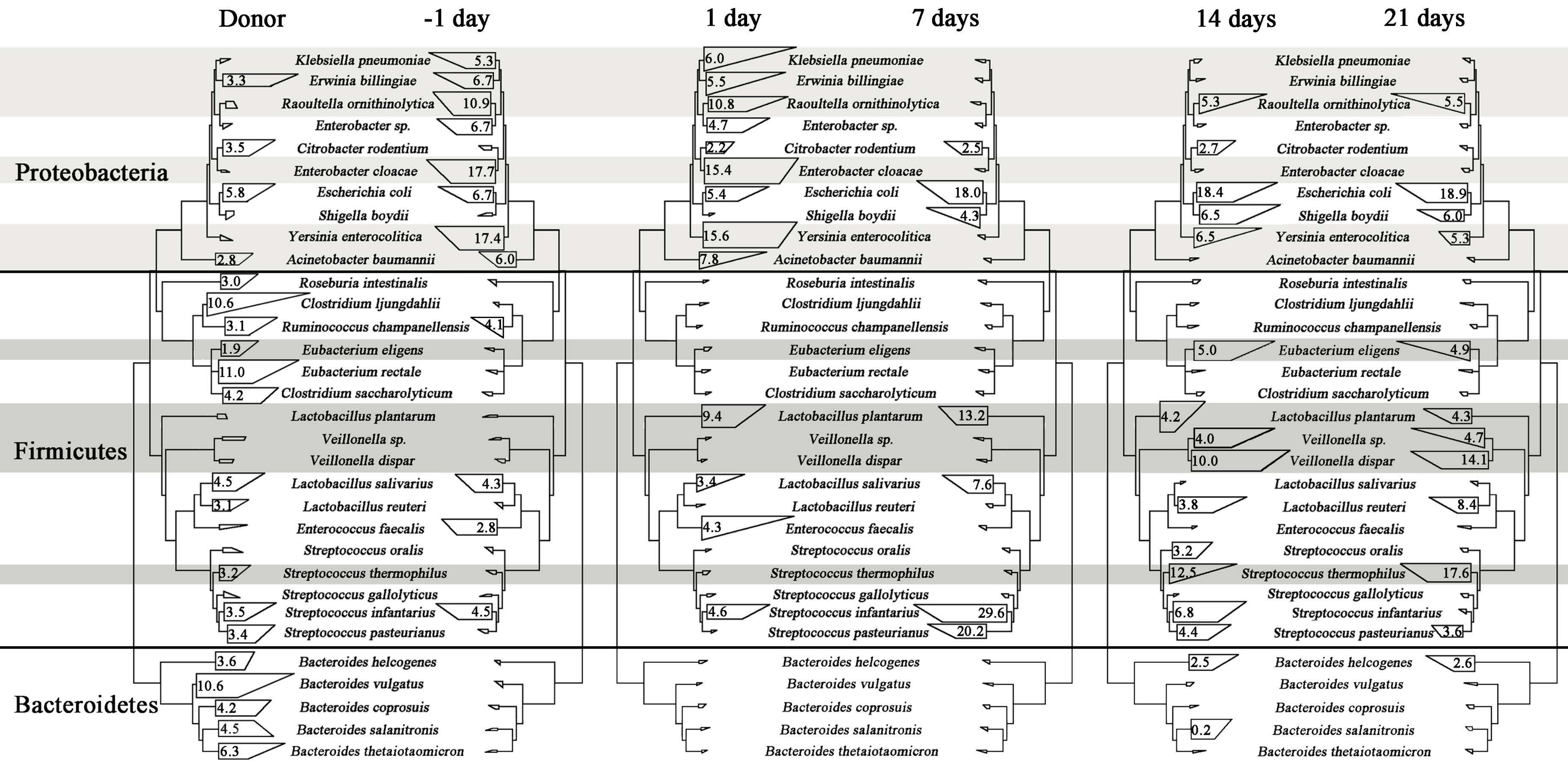
Donor
-1 day
1 day
7 days
14 days
21 days
Proteobacteria
Firmicutes
Bacteroidetes
Klebsiella pneumoniae
Erwinia billingiae
Raoultella ornithinolytica
Enterobacter sp.
Citrobacter rodentium
Enterobacter cloacae
Escherichia coli
Shigella boydii
Yersinia enterocolitica
Acinetobacter baumannii
Roseburia intestinalis
Clostridium ljungdahlii
Ruminococcus champanellensis
Eubacterium eligens
Eubacterium rectale
Clostridium saccharolyticum
Lactobacillus plantarum
Veillonella sp.
Veillonella dispar
Lactobacillus salivarius
Lactobacillus reuteri
Enterococcus faecalis
Streptococcus oralis
Streptococcus thermophilus
Streptococcus gallolyticus
Streptococcus infantarius
Streptococcus pasteurianus
Bacteroides helcogenes
Bacteroides vulgatus
Bacteroides coprosuis
Bacteroides salanitronis
Bacteroides thetaiotaomicron
