# Supplementary figures and images for: Successful treatment of severe sepsis and diarrhea after vagotomy utilizing fecal microbiota transplantation: a case report (part 2 of 2)
Source: Crit Care. 2015 Feb 9;19(1):37. doi: 10.1186/s13054-015-0738-7 (PMC4346118; doi:10.1186/s13054-015-0738-7)

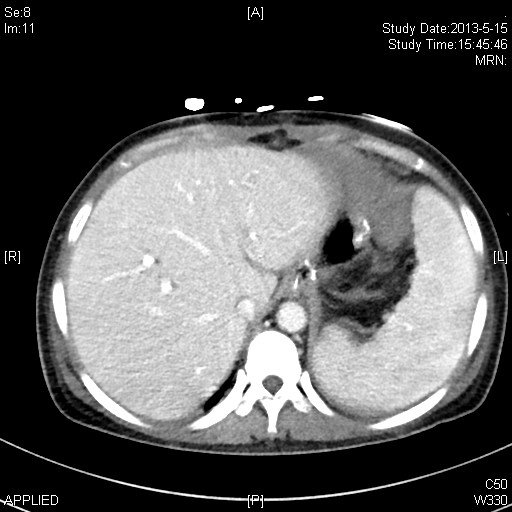

Supplement: Additional file 6: — Variations in phylogenetic distribution of 16S rDNA sequences following fecal infusion. The diagrammatic phylogenetic tree presents a summary of the rRNA sequences obtained from DGGE bands in this study. Phyla are named to the left of the tree, and lower taxonomic levels are given to the right. The number in the clade is designated as the relative proportion (%) in the whole fecal microbiota. [file 13054_2015_738_MOESM6_ESM.zip › CT images/CT images at 22 days after the surgery/Im11.jpg]

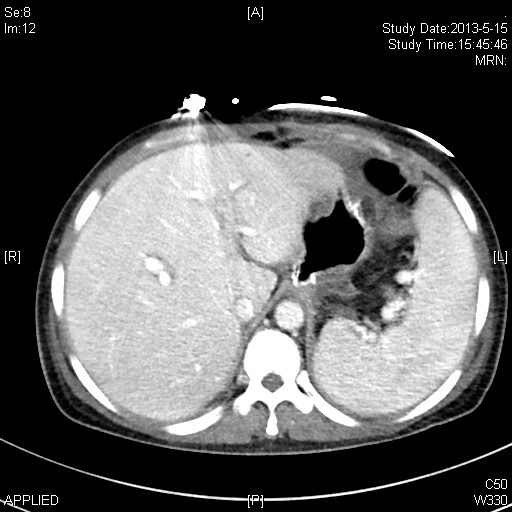

Supplement: Additional file 6: — Variations in phylogenetic distribution of 16S rDNA sequences following fecal infusion. The diagrammatic phylogenetic tree presents a summary of the rRNA sequences obtained from DGGE bands in this study. Phyla are named to the left of the tree, and lower taxonomic levels are given to the right. The number in the clade is designated as the relative proportion (%) in the whole fecal microbiota. [file 13054_2015_738_MOESM6_ESM.zip › CT images/CT images at 22 days after the surgery/Im12.jpg]

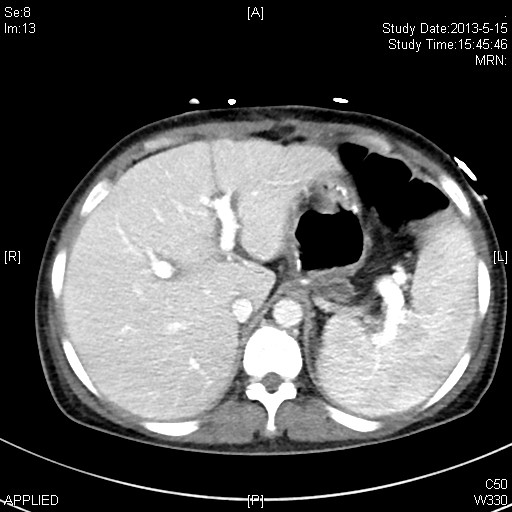

Supplement: Additional file 6: — Variations in phylogenetic distribution of 16S rDNA sequences following fecal infusion. The diagrammatic phylogenetic tree presents a summary of the rRNA sequences obtained from DGGE bands in this study. Phyla are named to the left of the tree, and lower taxonomic levels are given to the right. The number in the clade is designated as the relative proportion (%) in the whole fecal microbiota. [file 13054_2015_738_MOESM6_ESM.zip › CT images/CT images at 22 days after the surgery/Im13.jpg]

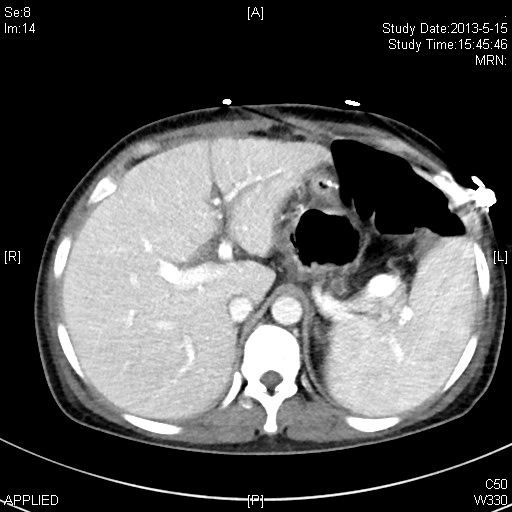

Supplement: Additional file 6: — Variations in phylogenetic distribution of 16S rDNA sequences following fecal infusion. The diagrammatic phylogenetic tree presents a summary of the rRNA sequences obtained from DGGE bands in this study. Phyla are named to the left of the tree, and lower taxonomic levels are given to the right. The number in the clade is designated as the relative proportion (%) in the whole fecal microbiota. [file 13054_2015_738_MOESM6_ESM.zip › CT images/CT images at 22 days after the surgery/Im14.jpg]

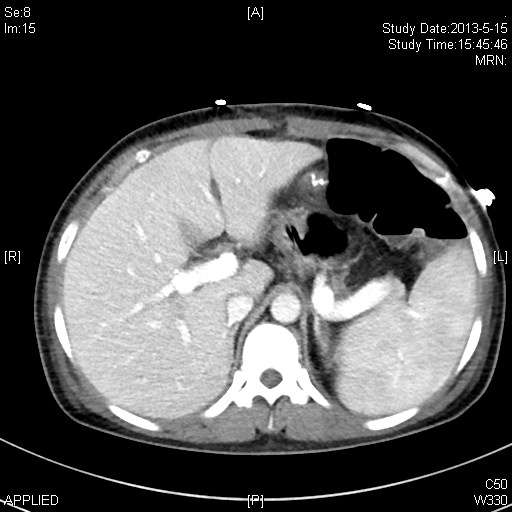

Supplement: Additional file 6: — Variations in phylogenetic distribution of 16S rDNA sequences following fecal infusion. The diagrammatic phylogenetic tree presents a summary of the rRNA sequences obtained from DGGE bands in this study. Phyla are named to the left of the tree, and lower taxonomic levels are given to the right. The number in the clade is designated as the relative proportion (%) in the whole fecal microbiota. [file 13054_2015_738_MOESM6_ESM.zip › CT images/CT images at 22 days after the surgery/Im15.jpg]

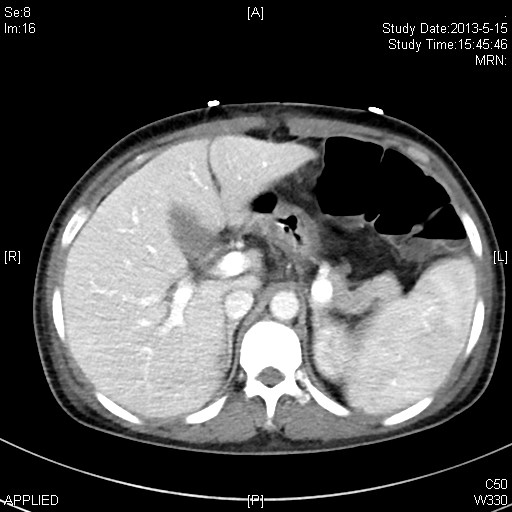

Supplement: Additional file 6: — Variations in phylogenetic distribution of 16S rDNA sequences following fecal infusion. The diagrammatic phylogenetic tree presents a summary of the rRNA sequences obtained from DGGE bands in this study. Phyla are named to the left of the tree, and lower taxonomic levels are given to the right. The number in the clade is designated as the relative proportion (%) in the whole fecal microbiota. [file 13054_2015_738_MOESM6_ESM.zip › CT images/CT images at 22 days after the surgery/Im16.jpg]

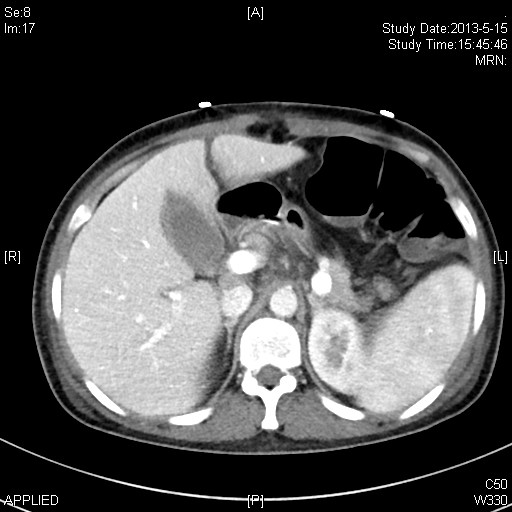

Supplement: Additional file 6: — Variations in phylogenetic distribution of 16S rDNA sequences following fecal infusion. The diagrammatic phylogenetic tree presents a summary of the rRNA sequences obtained from DGGE bands in this study. Phyla are named to the left of the tree, and lower taxonomic levels are given to the right. The number in the clade is designated as the relative proportion (%) in the whole fecal microbiota. [file 13054_2015_738_MOESM6_ESM.zip › CT images/CT images at 22 days after the surgery/Im17.jpg]

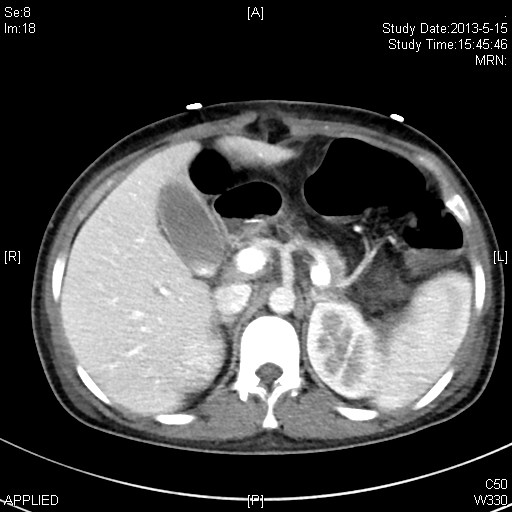

Supplement: Additional file 6: — Variations in phylogenetic distribution of 16S rDNA sequences following fecal infusion. The diagrammatic phylogenetic tree presents a summary of the rRNA sequences obtained from DGGE bands in this study. Phyla are named to the left of the tree, and lower taxonomic levels are given to the right. The number in the clade is designated as the relative proportion (%) in the whole fecal microbiota. [file 13054_2015_738_MOESM6_ESM.zip › CT images/CT images at 22 days after the surgery/Im18.jpg]

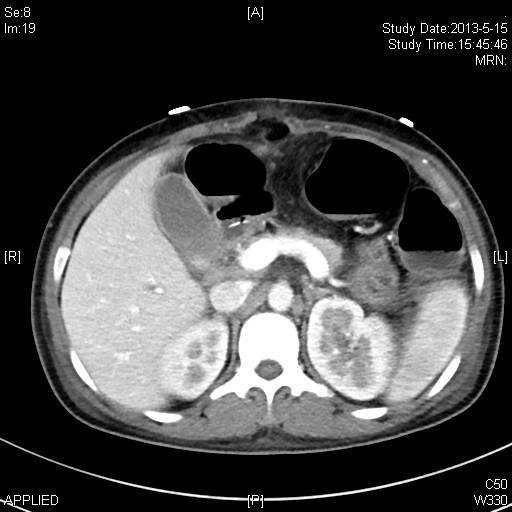

Supplement: Additional file 6: — Variations in phylogenetic distribution of 16S rDNA sequences following fecal infusion. The diagrammatic phylogenetic tree presents a summary of the rRNA sequences obtained from DGGE bands in this study. Phyla are named to the left of the tree, and lower taxonomic levels are given to the right. The number in the clade is designated as the relative proportion (%) in the whole fecal microbiota. [file 13054_2015_738_MOESM6_ESM.zip › CT images/CT images at 22 days after the surgery/Im19.jpg]

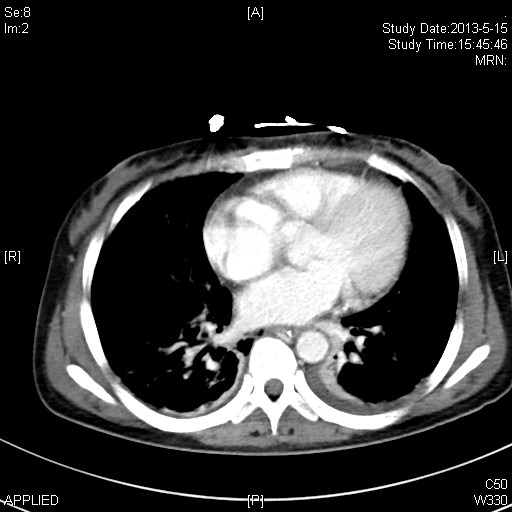

Supplement: Additional file 6: — Variations in phylogenetic distribution of 16S rDNA sequences following fecal infusion. The diagrammatic phylogenetic tree presents a summary of the rRNA sequences obtained from DGGE bands in this study. Phyla are named to the left of the tree, and lower taxonomic levels are given to the right. The number in the clade is designated as the relative proportion (%) in the whole fecal microbiota. [file 13054_2015_738_MOESM6_ESM.zip › CT images/CT images at 22 days after the surgery/Im2.jpg]

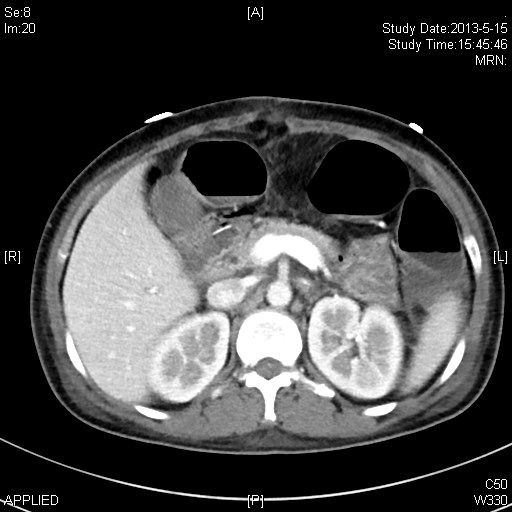

Supplement: Additional file 6: — Variations in phylogenetic distribution of 16S rDNA sequences following fecal infusion. The diagrammatic phylogenetic tree presents a summary of the rRNA sequences obtained from DGGE bands in this study. Phyla are named to the left of the tree, and lower taxonomic levels are given to the right. The number in the clade is designated as the relative proportion (%) in the whole fecal microbiota. [file 13054_2015_738_MOESM6_ESM.zip › CT images/CT images at 22 days after the surgery/Im20.jpg]

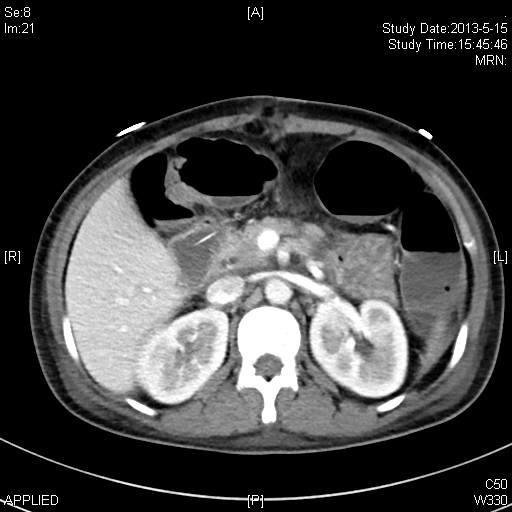

Supplement: Additional file 6: — Variations in phylogenetic distribution of 16S rDNA sequences following fecal infusion. The diagrammatic phylogenetic tree presents a summary of the rRNA sequences obtained from DGGE bands in this study. Phyla are named to the left of the tree, and lower taxonomic levels are given to the right. The number in the clade is designated as the relative proportion (%) in the whole fecal microbiota. [file 13054_2015_738_MOESM6_ESM.zip › CT images/CT images at 22 days after the surgery/Im21.jpg]

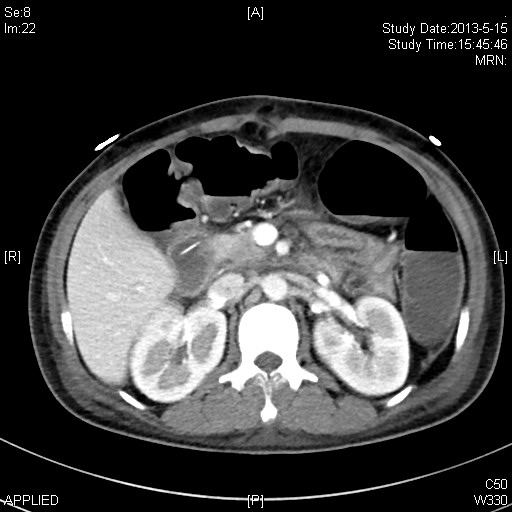

Supplement: Additional file 6: — Variations in phylogenetic distribution of 16S rDNA sequences following fecal infusion. The diagrammatic phylogenetic tree presents a summary of the rRNA sequences obtained from DGGE bands in this study. Phyla are named to the left of the tree, and lower taxonomic levels are given to the right. The number in the clade is designated as the relative proportion (%) in the whole fecal microbiota. [file 13054_2015_738_MOESM6_ESM.zip › CT images/CT images at 22 days after the surgery/Im22.jpg]

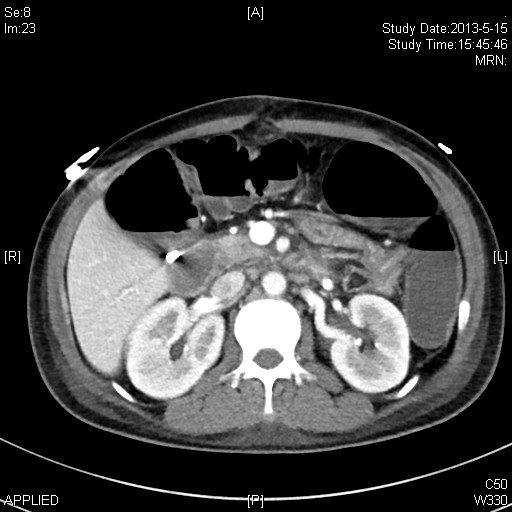

Supplement: Additional file 6: — Variations in phylogenetic distribution of 16S rDNA sequences following fecal infusion. The diagrammatic phylogenetic tree presents a summary of the rRNA sequences obtained from DGGE bands in this study. Phyla are named to the left of the tree, and lower taxonomic levels are given to the right. The number in the clade is designated as the relative proportion (%) in the whole fecal microbiota. [file 13054_2015_738_MOESM6_ESM.zip › CT images/CT images at 22 days after the surgery/Im23.jpg]

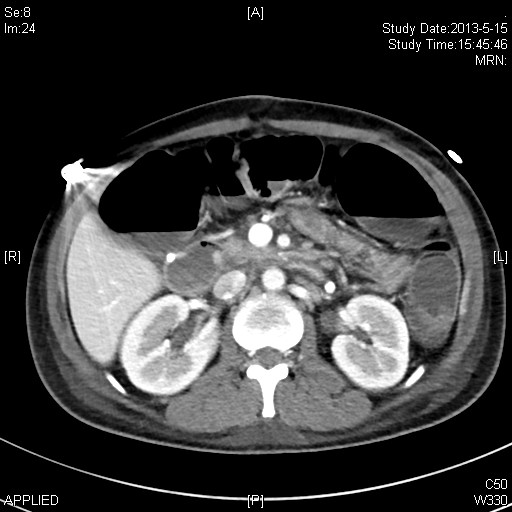

Supplement: Additional file 6: — Variations in phylogenetic distribution of 16S rDNA sequences following fecal infusion. The diagrammatic phylogenetic tree presents a summary of the rRNA sequences obtained from DGGE bands in this study. Phyla are named to the left of the tree, and lower taxonomic levels are given to the right. The number in the clade is designated as the relative proportion (%) in the whole fecal microbiota. [file 13054_2015_738_MOESM6_ESM.zip › CT images/CT images at 22 days after the surgery/Im24.jpg]

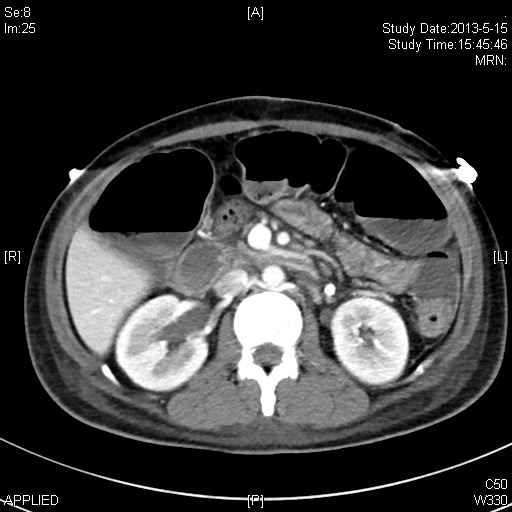

Supplement: Additional file 6: — Variations in phylogenetic distribution of 16S rDNA sequences following fecal infusion. The diagrammatic phylogenetic tree presents a summary of the rRNA sequences obtained from DGGE bands in this study. Phyla are named to the left of the tree, and lower taxonomic levels are given to the right. The number in the clade is designated as the relative proportion (%) in the whole fecal microbiota. [file 13054_2015_738_MOESM6_ESM.zip › CT images/CT images at 22 days after the surgery/Im25.jpg]

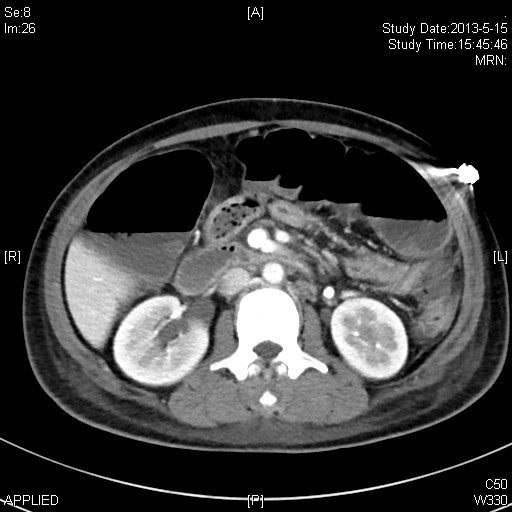

Supplement: Additional file 6: — Variations in phylogenetic distribution of 16S rDNA sequences following fecal infusion. The diagrammatic phylogenetic tree presents a summary of the rRNA sequences obtained from DGGE bands in this study. Phyla are named to the left of the tree, and lower taxonomic levels are given to the right. The number in the clade is designated as the relative proportion (%) in the whole fecal microbiota. [file 13054_2015_738_MOESM6_ESM.zip › CT images/CT images at 22 days after the surgery/Im26.jpg]

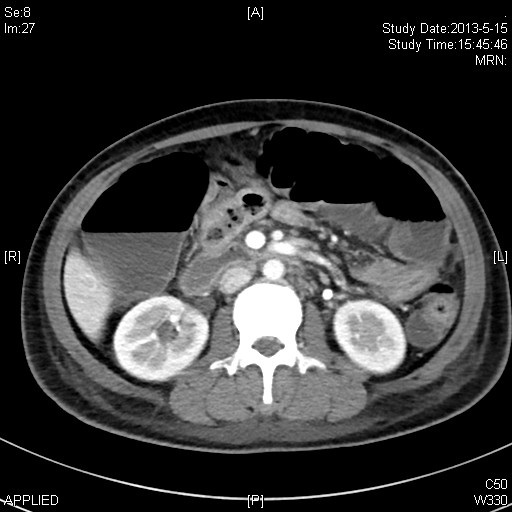

Supplement: Additional file 6: — Variations in phylogenetic distribution of 16S rDNA sequences following fecal infusion. The diagrammatic phylogenetic tree presents a summary of the rRNA sequences obtained from DGGE bands in this study. Phyla are named to the left of the tree, and lower taxonomic levels are given to the right. The number in the clade is designated as the relative proportion (%) in the whole fecal microbiota. [file 13054_2015_738_MOESM6_ESM.zip › CT images/CT images at 22 days after the surgery/Im27.jpg]

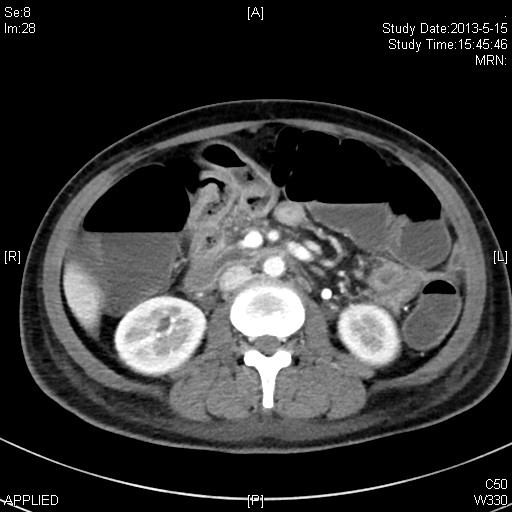

Supplement: Additional file 6: — Variations in phylogenetic distribution of 16S rDNA sequences following fecal infusion. The diagrammatic phylogenetic tree presents a summary of the rRNA sequences obtained from DGGE bands in this study. Phyla are named to the left of the tree, and lower taxonomic levels are given to the right. The number in the clade is designated as the relative proportion (%) in the whole fecal microbiota. [file 13054_2015_738_MOESM6_ESM.zip › CT images/CT images at 22 days after the surgery/Im28.jpg]

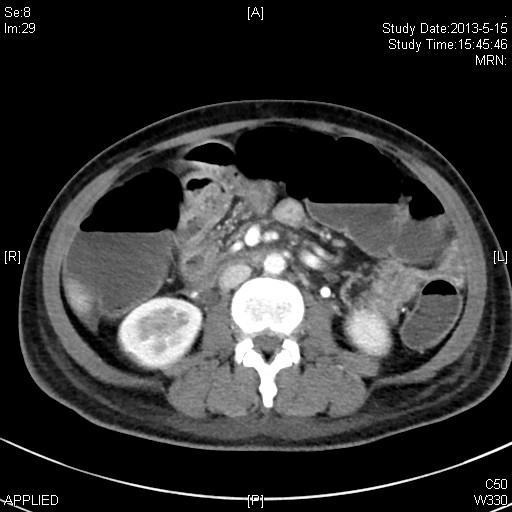

Supplement: Additional file 6: — Variations in phylogenetic distribution of 16S rDNA sequences following fecal infusion. The diagrammatic phylogenetic tree presents a summary of the rRNA sequences obtained from DGGE bands in this study. Phyla are named to the left of the tree, and lower taxonomic levels are given to the right. The number in the clade is designated as the relative proportion (%) in the whole fecal microbiota. [file 13054_2015_738_MOESM6_ESM.zip › CT images/CT images at 22 days after the surgery/Im29.jpg]

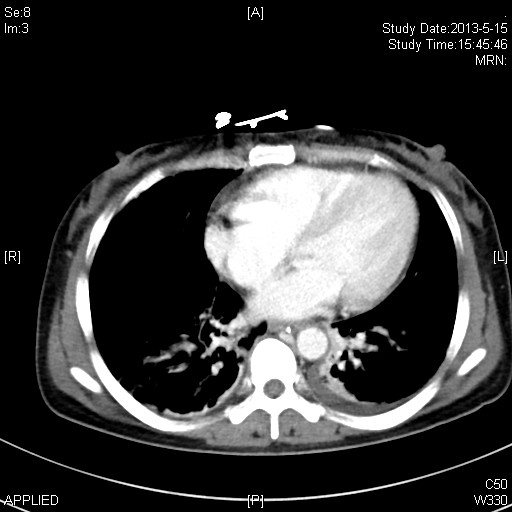

Supplement: Additional file 6: — Variations in phylogenetic distribution of 16S rDNA sequences following fecal infusion. The diagrammatic phylogenetic tree presents a summary of the rRNA sequences obtained from DGGE bands in this study. Phyla are named to the left of the tree, and lower taxonomic levels are given to the right. The number in the clade is designated as the relative proportion (%) in the whole fecal microbiota. [file 13054_2015_738_MOESM6_ESM.zip › CT images/CT images at 22 days after the surgery/Im3.jpg]

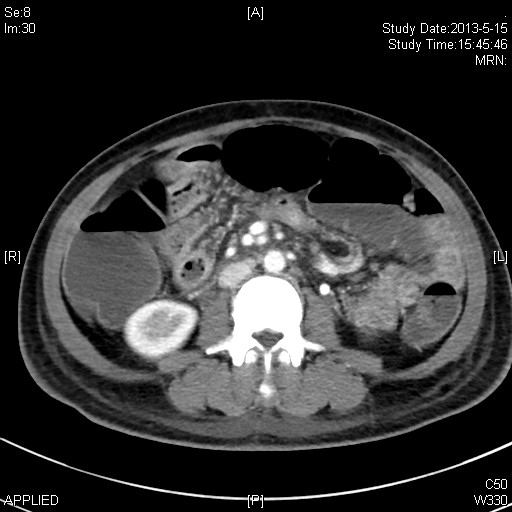

Supplement: Additional file 6: — Variations in phylogenetic distribution of 16S rDNA sequences following fecal infusion. The diagrammatic phylogenetic tree presents a summary of the rRNA sequences obtained from DGGE bands in this study. Phyla are named to the left of the tree, and lower taxonomic levels are given to the right. The number in the clade is designated as the relative proportion (%) in the whole fecal microbiota. [file 13054_2015_738_MOESM6_ESM.zip › CT images/CT images at 22 days after the surgery/Im30.jpg]

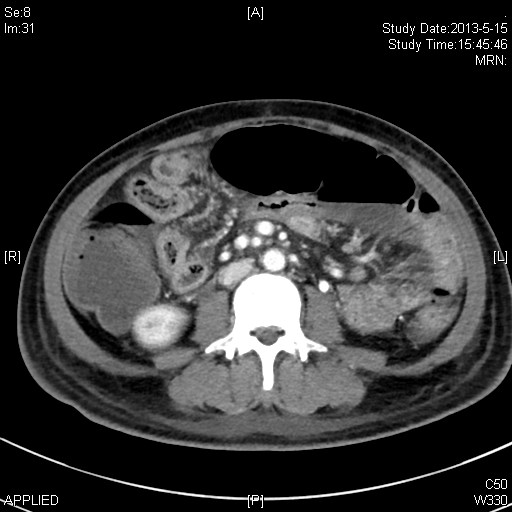

Supplement: Additional file 6: — Variations in phylogenetic distribution of 16S rDNA sequences following fecal infusion. The diagrammatic phylogenetic tree presents a summary of the rRNA sequences obtained from DGGE bands in this study. Phyla are named to the left of the tree, and lower taxonomic levels are given to the right. The number in the clade is designated as the relative proportion (%) in the whole fecal microbiota. [file 13054_2015_738_MOESM6_ESM.zip › CT images/CT images at 22 days after the surgery/Im31.jpg]

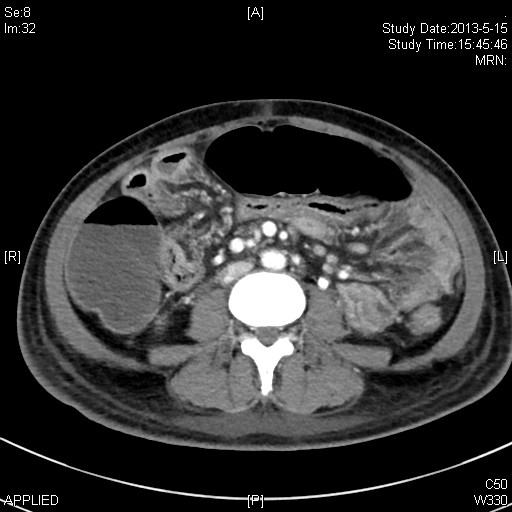

Supplement: Additional file 6: — Variations in phylogenetic distribution of 16S rDNA sequences following fecal infusion. The diagrammatic phylogenetic tree presents a summary of the rRNA sequences obtained from DGGE bands in this study. Phyla are named to the left of the tree, and lower taxonomic levels are given to the right. The number in the clade is designated as the relative proportion (%) in the whole fecal microbiota. [file 13054_2015_738_MOESM6_ESM.zip › CT images/CT images at 22 days after the surgery/Im32.jpg]

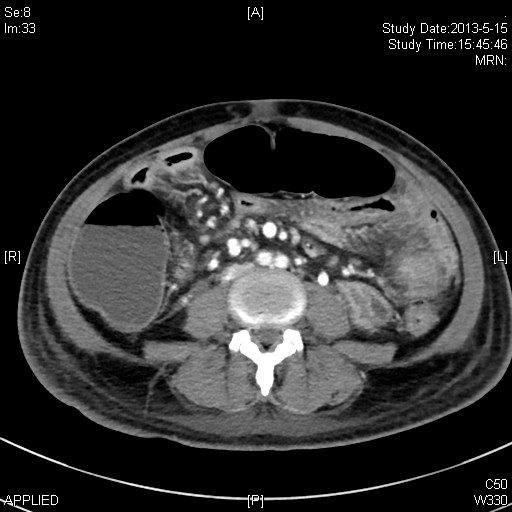

Supplement: Additional file 6: — Variations in phylogenetic distribution of 16S rDNA sequences following fecal infusion. The diagrammatic phylogenetic tree presents a summary of the rRNA sequences obtained from DGGE bands in this study. Phyla are named to the left of the tree, and lower taxonomic levels are given to the right. The number in the clade is designated as the relative proportion (%) in the whole fecal microbiota. [file 13054_2015_738_MOESM6_ESM.zip › CT images/CT images at 22 days after the surgery/Im33.jpg]

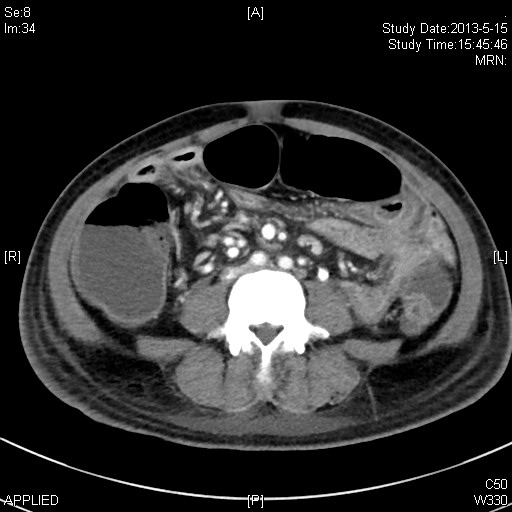

Supplement: Additional file 6: — Variations in phylogenetic distribution of 16S rDNA sequences following fecal infusion. The diagrammatic phylogenetic tree presents a summary of the rRNA sequences obtained from DGGE bands in this study. Phyla are named to the left of the tree, and lower taxonomic levels are given to the right. The number in the clade is designated as the relative proportion (%) in the whole fecal microbiota. [file 13054_2015_738_MOESM6_ESM.zip › CT images/CT images at 22 days after the surgery/Im34.jpg]

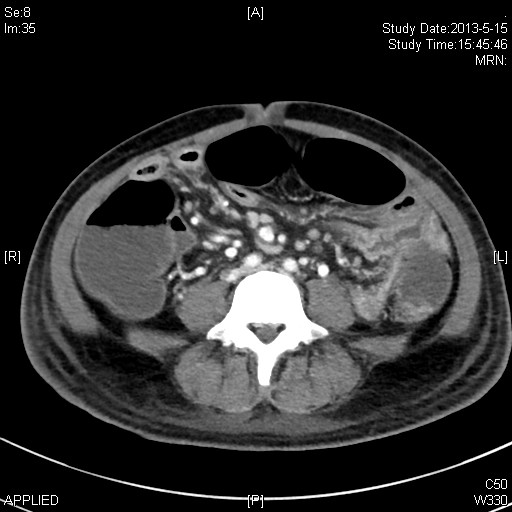

Supplement: Additional file 6: — Variations in phylogenetic distribution of 16S rDNA sequences following fecal infusion. The diagrammatic phylogenetic tree presents a summary of the rRNA sequences obtained from DGGE bands in this study. Phyla are named to the left of the tree, and lower taxonomic levels are given to the right. The number in the clade is designated as the relative proportion (%) in the whole fecal microbiota. [file 13054_2015_738_MOESM6_ESM.zip › CT images/CT images at 22 days after the surgery/Im35.jpg]

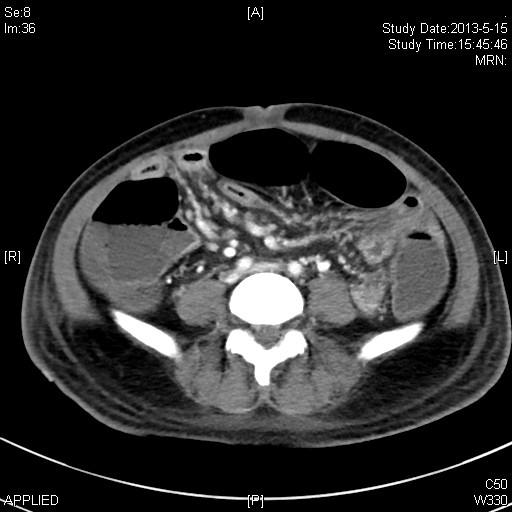

Supplement: Additional file 6: — Variations in phylogenetic distribution of 16S rDNA sequences following fecal infusion. The diagrammatic phylogenetic tree presents a summary of the rRNA sequences obtained from DGGE bands in this study. Phyla are named to the left of the tree, and lower taxonomic levels are given to the right. The number in the clade is designated as the relative proportion (%) in the whole fecal microbiota. [file 13054_2015_738_MOESM6_ESM.zip › CT images/CT images at 22 days after the surgery/Im36.jpg]

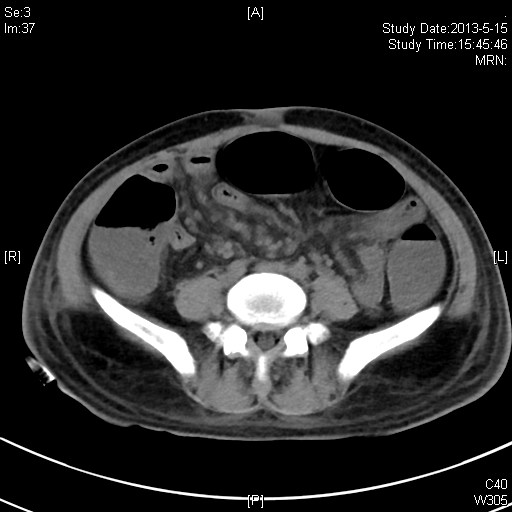

Supplement: Additional file 6: — Variations in phylogenetic distribution of 16S rDNA sequences following fecal infusion. The diagrammatic phylogenetic tree presents a summary of the rRNA sequences obtained from DGGE bands in this study. Phyla are named to the left of the tree, and lower taxonomic levels are given to the right. The number in the clade is designated as the relative proportion (%) in the whole fecal microbiota. [file 13054_2015_738_MOESM6_ESM.zip › CT images/CT images at 22 days after the surgery/Im37.jpg]

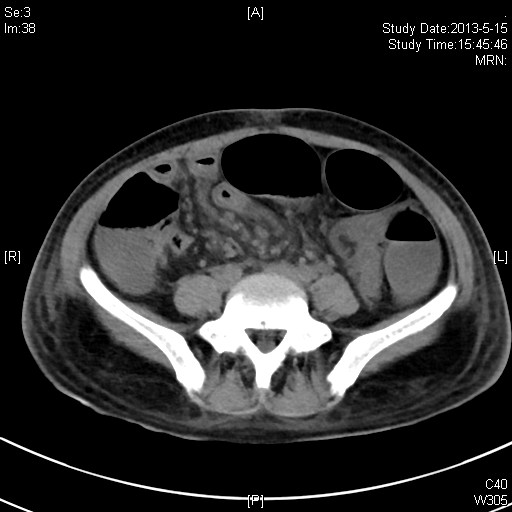

Supplement: Additional file 6: — Variations in phylogenetic distribution of 16S rDNA sequences following fecal infusion. The diagrammatic phylogenetic tree presents a summary of the rRNA sequences obtained from DGGE bands in this study. Phyla are named to the left of the tree, and lower taxonomic levels are given to the right. The number in the clade is designated as the relative proportion (%) in the whole fecal microbiota. [file 13054_2015_738_MOESM6_ESM.zip › CT images/CT images at 22 days after the surgery/Im38.jpg]

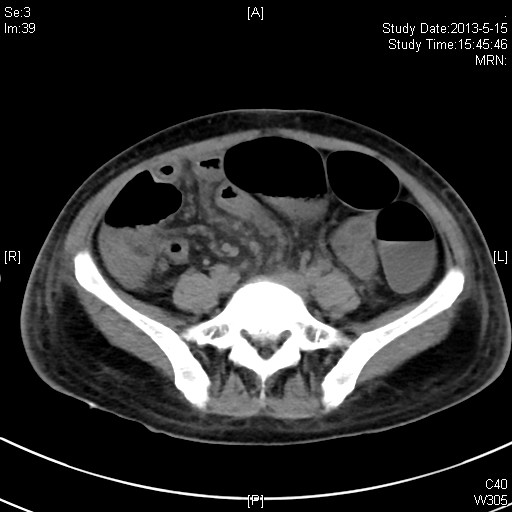

Supplement: Additional file 6: — Variations in phylogenetic distribution of 16S rDNA sequences following fecal infusion. The diagrammatic phylogenetic tree presents a summary of the rRNA sequences obtained from DGGE bands in this study. Phyla are named to the left of the tree, and lower taxonomic levels are given to the right. The number in the clade is designated as the relative proportion (%) in the whole fecal microbiota. [file 13054_2015_738_MOESM6_ESM.zip › CT images/CT images at 22 days after the surgery/Im39.jpg]

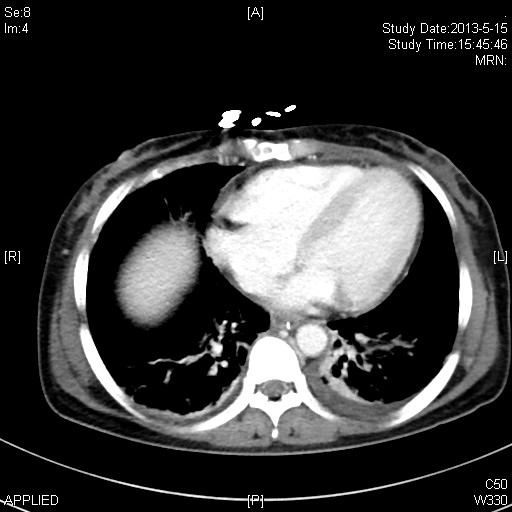

Supplement: Additional file 6: — Variations in phylogenetic distribution of 16S rDNA sequences following fecal infusion. The diagrammatic phylogenetic tree presents a summary of the rRNA sequences obtained from DGGE bands in this study. Phyla are named to the left of the tree, and lower taxonomic levels are given to the right. The number in the clade is designated as the relative proportion (%) in the whole fecal microbiota. [file 13054_2015_738_MOESM6_ESM.zip › CT images/CT images at 22 days after the surgery/Im4.jpg]

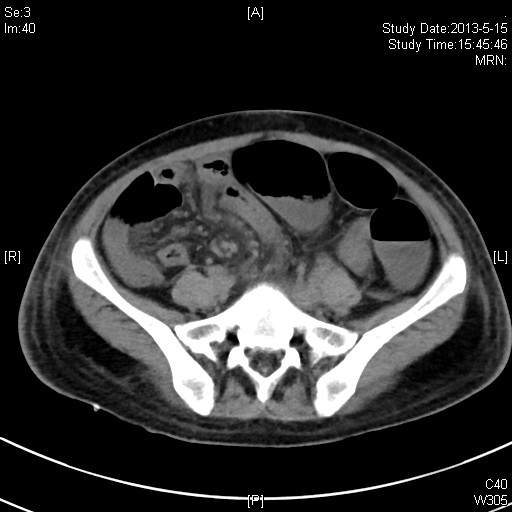

Supplement: Additional file 6: — Variations in phylogenetic distribution of 16S rDNA sequences following fecal infusion. The diagrammatic phylogenetic tree presents a summary of the rRNA sequences obtained from DGGE bands in this study. Phyla are named to the left of the tree, and lower taxonomic levels are given to the right. The number in the clade is designated as the relative proportion (%) in the whole fecal microbiota. [file 13054_2015_738_MOESM6_ESM.zip › CT images/CT images at 22 days after the surgery/Im40.jpg]

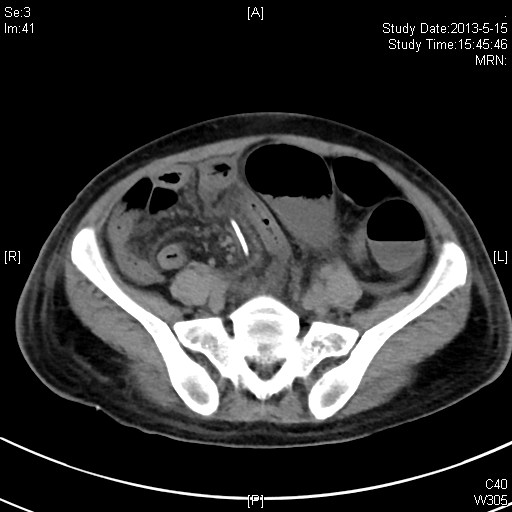

Supplement: Additional file 6: — Variations in phylogenetic distribution of 16S rDNA sequences following fecal infusion. The diagrammatic phylogenetic tree presents a summary of the rRNA sequences obtained from DGGE bands in this study. Phyla are named to the left of the tree, and lower taxonomic levels are given to the right. The number in the clade is designated as the relative proportion (%) in the whole fecal microbiota. [file 13054_2015_738_MOESM6_ESM.zip › CT images/CT images at 22 days after the surgery/Im41.jpg]

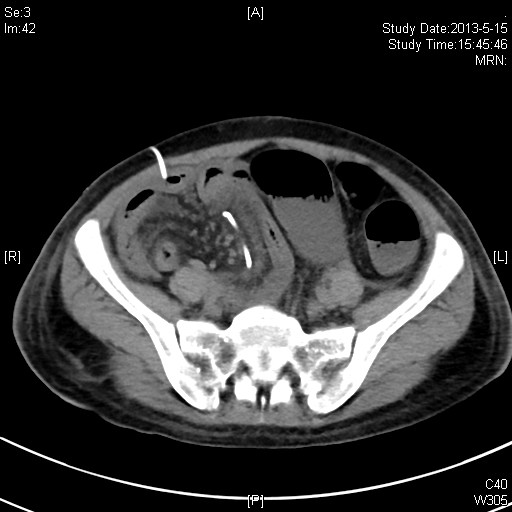

Supplement: Additional file 6: — Variations in phylogenetic distribution of 16S rDNA sequences following fecal infusion. The diagrammatic phylogenetic tree presents a summary of the rRNA sequences obtained from DGGE bands in this study. Phyla are named to the left of the tree, and lower taxonomic levels are given to the right. The number in the clade is designated as the relative proportion (%) in the whole fecal microbiota. [file 13054_2015_738_MOESM6_ESM.zip › CT images/CT images at 22 days after the surgery/Im42.jpg]

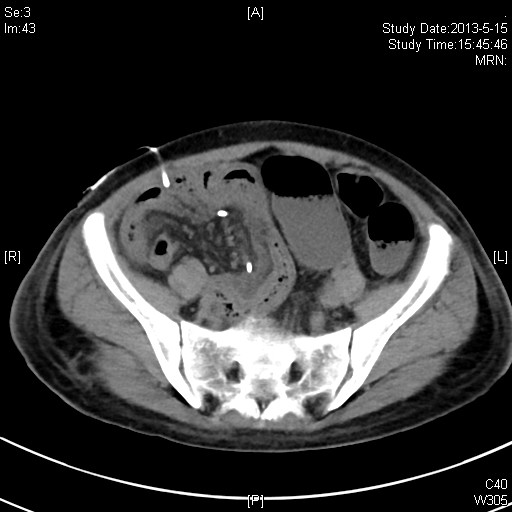

Supplement: Additional file 6: — Variations in phylogenetic distribution of 16S rDNA sequences following fecal infusion. The diagrammatic phylogenetic tree presents a summary of the rRNA sequences obtained from DGGE bands in this study. Phyla are named to the left of the tree, and lower taxonomic levels are given to the right. The number in the clade is designated as the relative proportion (%) in the whole fecal microbiota. [file 13054_2015_738_MOESM6_ESM.zip › CT images/CT images at 22 days after the surgery/Im43.jpg]

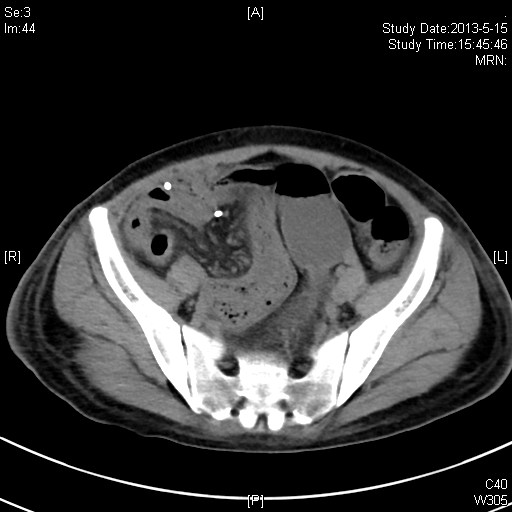

Supplement: Additional file 6: — Variations in phylogenetic distribution of 16S rDNA sequences following fecal infusion. The diagrammatic phylogenetic tree presents a summary of the rRNA sequences obtained from DGGE bands in this study. Phyla are named to the left of the tree, and lower taxonomic levels are given to the right. The number in the clade is designated as the relative proportion (%) in the whole fecal microbiota. [file 13054_2015_738_MOESM6_ESM.zip › CT images/CT images at 22 days after the surgery/Im44.jpg]

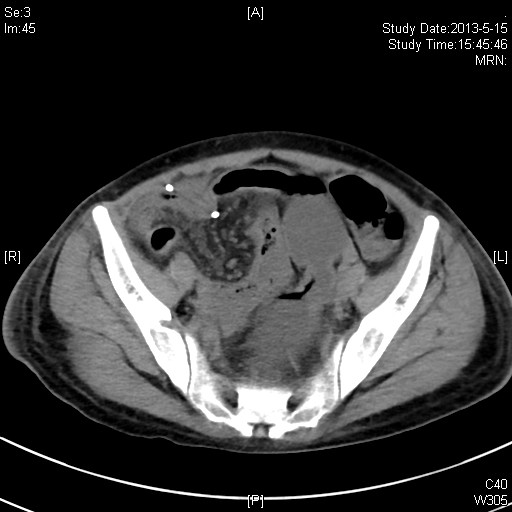

Supplement: Additional file 6: — Variations in phylogenetic distribution of 16S rDNA sequences following fecal infusion. The diagrammatic phylogenetic tree presents a summary of the rRNA sequences obtained from DGGE bands in this study. Phyla are named to the left of the tree, and lower taxonomic levels are given to the right. The number in the clade is designated as the relative proportion (%) in the whole fecal microbiota. [file 13054_2015_738_MOESM6_ESM.zip › CT images/CT images at 22 days after the surgery/Im45.jpg]

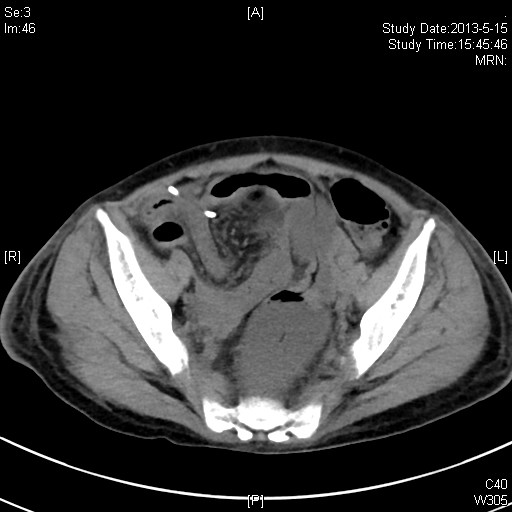

Supplement: Additional file 6: — Variations in phylogenetic distribution of 16S rDNA sequences following fecal infusion. The diagrammatic phylogenetic tree presents a summary of the rRNA sequences obtained from DGGE bands in this study. Phyla are named to the left of the tree, and lower taxonomic levels are given to the right. The number in the clade is designated as the relative proportion (%) in the whole fecal microbiota. [file 13054_2015_738_MOESM6_ESM.zip › CT images/CT images at 22 days after the surgery/Im46.jpg]

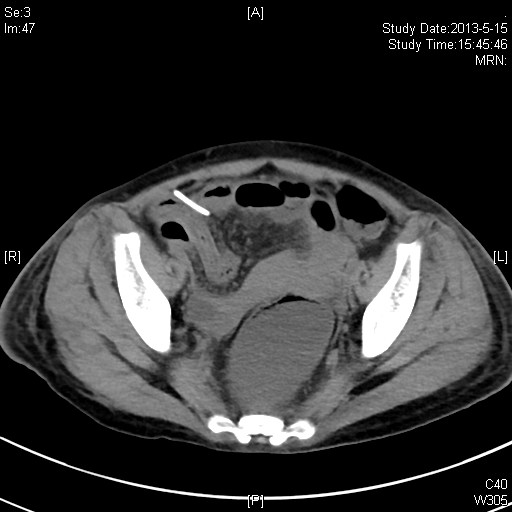

Supplement: Additional file 6: — Variations in phylogenetic distribution of 16S rDNA sequences following fecal infusion. The diagrammatic phylogenetic tree presents a summary of the rRNA sequences obtained from DGGE bands in this study. Phyla are named to the left of the tree, and lower taxonomic levels are given to the right. The number in the clade is designated as the relative proportion (%) in the whole fecal microbiota. [file 13054_2015_738_MOESM6_ESM.zip › CT images/CT images at 22 days after the surgery/Im47.jpg]

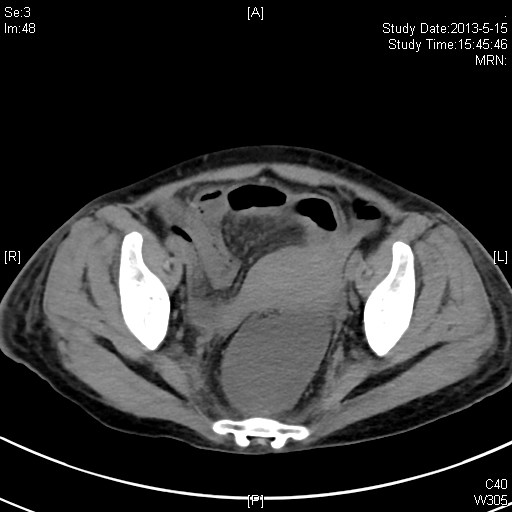

Supplement: Additional file 6: — Variations in phylogenetic distribution of 16S rDNA sequences following fecal infusion. The diagrammatic phylogenetic tree presents a summary of the rRNA sequences obtained from DGGE bands in this study. Phyla are named to the left of the tree, and lower taxonomic levels are given to the right. The number in the clade is designated as the relative proportion (%) in the whole fecal microbiota. [file 13054_2015_738_MOESM6_ESM.zip › CT images/CT images at 22 days after the surgery/Im48.jpg]

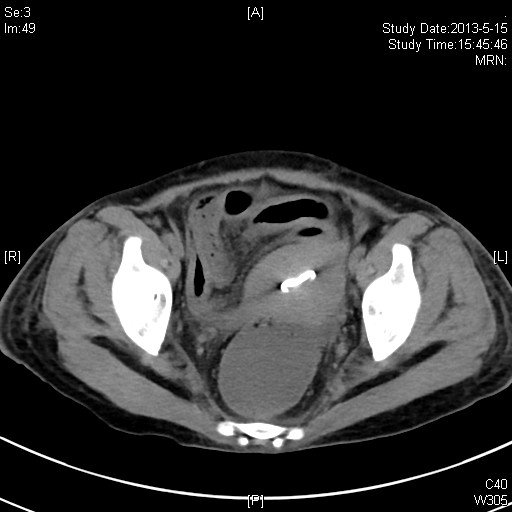

Supplement: Additional file 6: — Variations in phylogenetic distribution of 16S rDNA sequences following fecal infusion. The diagrammatic phylogenetic tree presents a summary of the rRNA sequences obtained from DGGE bands in this study. Phyla are named to the left of the tree, and lower taxonomic levels are given to the right. The number in the clade is designated as the relative proportion (%) in the whole fecal microbiota. [file 13054_2015_738_MOESM6_ESM.zip › CT images/CT images at 22 days after the surgery/Im49.jpg]

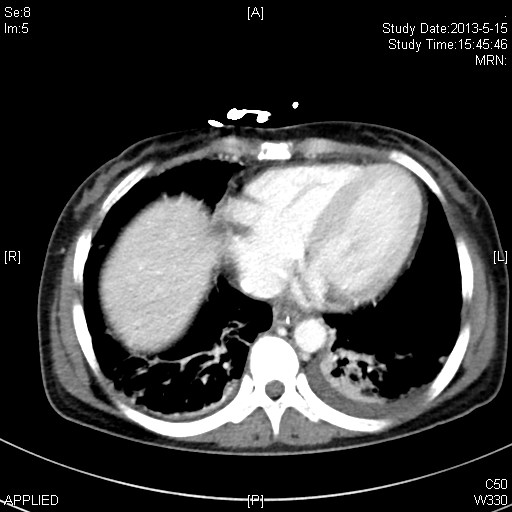

Supplement: Additional file 6: — Variations in phylogenetic distribution of 16S rDNA sequences following fecal infusion. The diagrammatic phylogenetic tree presents a summary of the rRNA sequences obtained from DGGE bands in this study. Phyla are named to the left of the tree, and lower taxonomic levels are given to the right. The number in the clade is designated as the relative proportion (%) in the whole fecal microbiota. [file 13054_2015_738_MOESM6_ESM.zip › CT images/CT images at 22 days after the surgery/Im5.jpg]

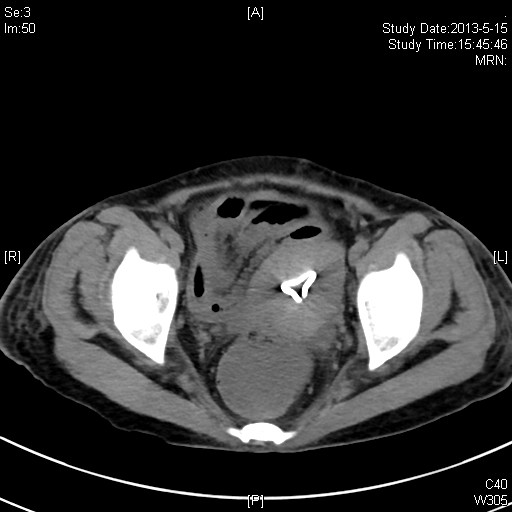

Supplement: Additional file 6: — Variations in phylogenetic distribution of 16S rDNA sequences following fecal infusion. The diagrammatic phylogenetic tree presents a summary of the rRNA sequences obtained from DGGE bands in this study. Phyla are named to the left of the tree, and lower taxonomic levels are given to the right. The number in the clade is designated as the relative proportion (%) in the whole fecal microbiota. [file 13054_2015_738_MOESM6_ESM.zip › CT images/CT images at 22 days after the surgery/Im50.jpg]

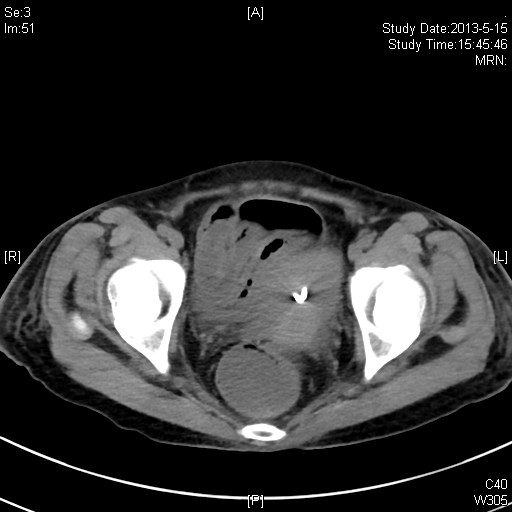

Supplement: Additional file 6: — Variations in phylogenetic distribution of 16S rDNA sequences following fecal infusion. The diagrammatic phylogenetic tree presents a summary of the rRNA sequences obtained from DGGE bands in this study. Phyla are named to the left of the tree, and lower taxonomic levels are given to the right. The number in the clade is designated as the relative proportion (%) in the whole fecal microbiota. [file 13054_2015_738_MOESM6_ESM.zip › CT images/CT images at 22 days after the surgery/Im51.jpg]

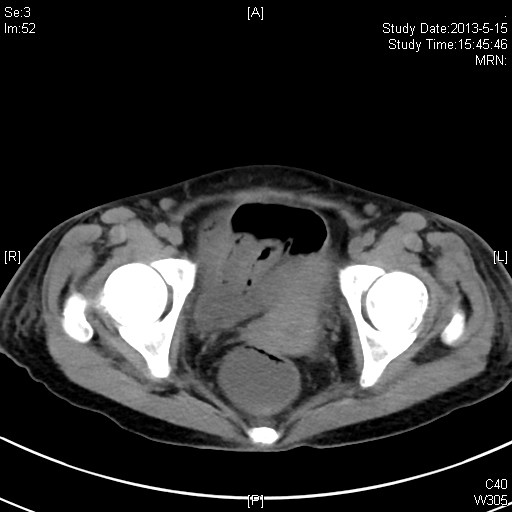

Supplement: Additional file 6: — Variations in phylogenetic distribution of 16S rDNA sequences following fecal infusion. The diagrammatic phylogenetic tree presents a summary of the rRNA sequences obtained from DGGE bands in this study. Phyla are named to the left of the tree, and lower taxonomic levels are given to the right. The number in the clade is designated as the relative proportion (%) in the whole fecal microbiota. [file 13054_2015_738_MOESM6_ESM.zip › CT images/CT images at 22 days after the surgery/Im52.jpg]

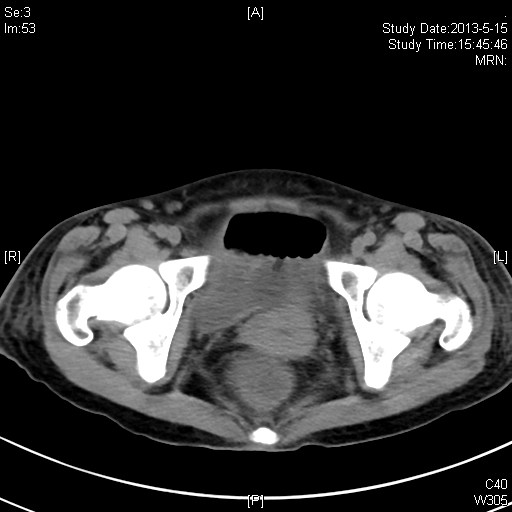

Supplement: Additional file 6: — Variations in phylogenetic distribution of 16S rDNA sequences following fecal infusion. The diagrammatic phylogenetic tree presents a summary of the rRNA sequences obtained from DGGE bands in this study. Phyla are named to the left of the tree, and lower taxonomic levels are given to the right. The number in the clade is designated as the relative proportion (%) in the whole fecal microbiota. [file 13054_2015_738_MOESM6_ESM.zip › CT images/CT images at 22 days after the surgery/Im53.jpg]

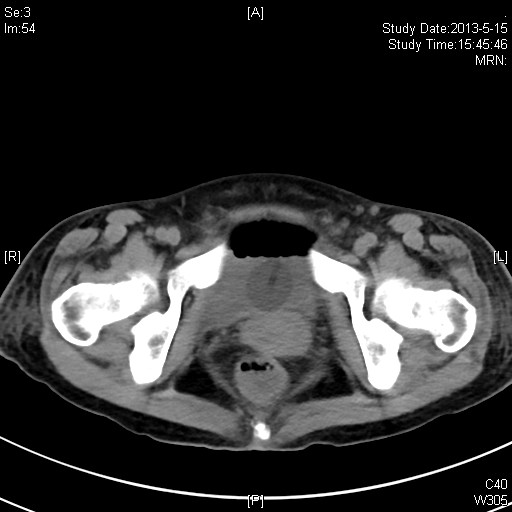

Supplement: Additional file 6: — Variations in phylogenetic distribution of 16S rDNA sequences following fecal infusion. The diagrammatic phylogenetic tree presents a summary of the rRNA sequences obtained from DGGE bands in this study. Phyla are named to the left of the tree, and lower taxonomic levels are given to the right. The number in the clade is designated as the relative proportion (%) in the whole fecal microbiota. [file 13054_2015_738_MOESM6_ESM.zip › CT images/CT images at 22 days after the surgery/Im54.jpg]

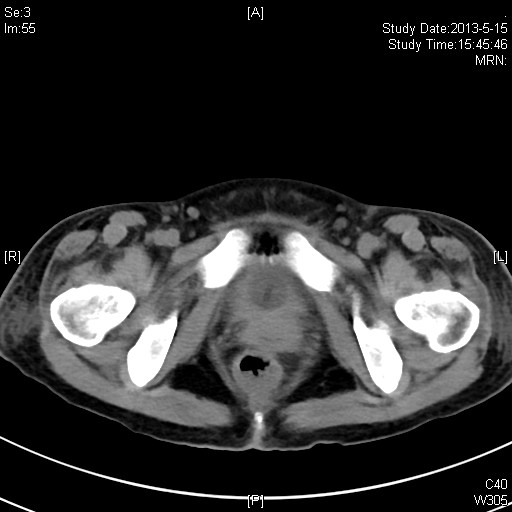

Supplement: Additional file 6: — Variations in phylogenetic distribution of 16S rDNA sequences following fecal infusion. The diagrammatic phylogenetic tree presents a summary of the rRNA sequences obtained from DGGE bands in this study. Phyla are named to the left of the tree, and lower taxonomic levels are given to the right. The number in the clade is designated as the relative proportion (%) in the whole fecal microbiota. [file 13054_2015_738_MOESM6_ESM.zip › CT images/CT images at 22 days after the surgery/Im55.jpg]

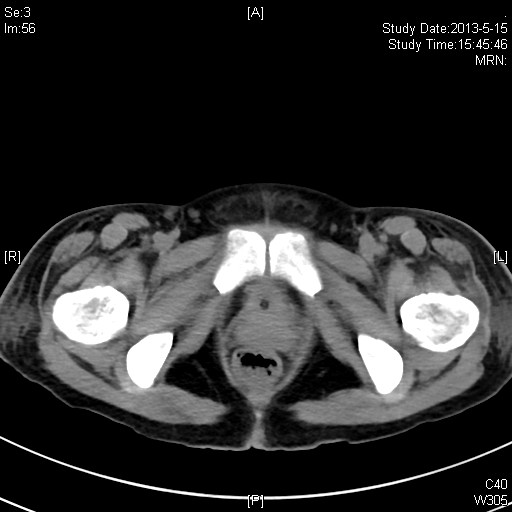

Supplement: Additional file 6: — Variations in phylogenetic distribution of 16S rDNA sequences following fecal infusion. The diagrammatic phylogenetic tree presents a summary of the rRNA sequences obtained from DGGE bands in this study. Phyla are named to the left of the tree, and lower taxonomic levels are given to the right. The number in the clade is designated as the relative proportion (%) in the whole fecal microbiota. [file 13054_2015_738_MOESM6_ESM.zip › CT images/CT images at 22 days after the surgery/Im56.jpg]

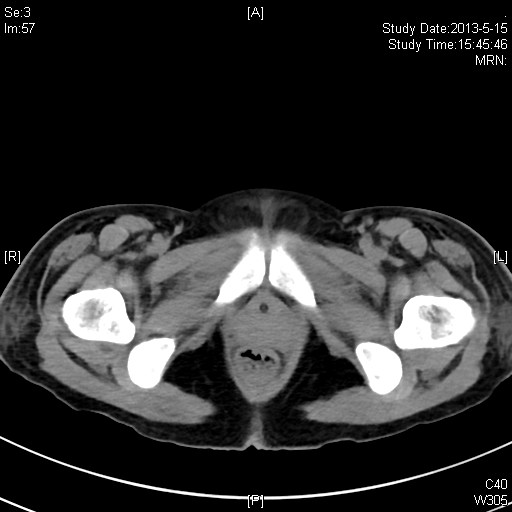

Supplement: Additional file 6: — Variations in phylogenetic distribution of 16S rDNA sequences following fecal infusion. The diagrammatic phylogenetic tree presents a summary of the rRNA sequences obtained from DGGE bands in this study. Phyla are named to the left of the tree, and lower taxonomic levels are given to the right. The number in the clade is designated as the relative proportion (%) in the whole fecal microbiota. [file 13054_2015_738_MOESM6_ESM.zip › CT images/CT images at 22 days after the surgery/Im57.jpg]

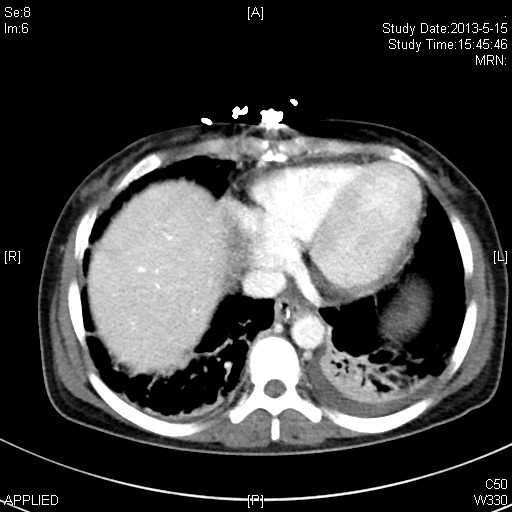

Supplement: Additional file 6: — Variations in phylogenetic distribution of 16S rDNA sequences following fecal infusion. The diagrammatic phylogenetic tree presents a summary of the rRNA sequences obtained from DGGE bands in this study. Phyla are named to the left of the tree, and lower taxonomic levels are given to the right. The number in the clade is designated as the relative proportion (%) in the whole fecal microbiota. [file 13054_2015_738_MOESM6_ESM.zip › CT images/CT images at 22 days after the surgery/Im6.jpg]

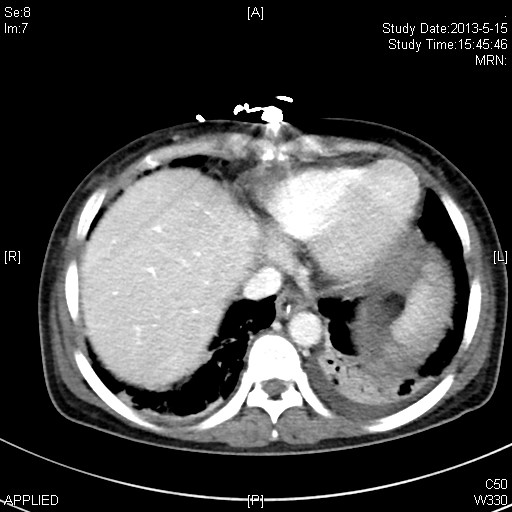

Supplement: Additional file 6: — Variations in phylogenetic distribution of 16S rDNA sequences following fecal infusion. The diagrammatic phylogenetic tree presents a summary of the rRNA sequences obtained from DGGE bands in this study. Phyla are named to the left of the tree, and lower taxonomic levels are given to the right. The number in the clade is designated as the relative proportion (%) in the whole fecal microbiota. [file 13054_2015_738_MOESM6_ESM.zip › CT images/CT images at 22 days after the surgery/Im7.jpg]

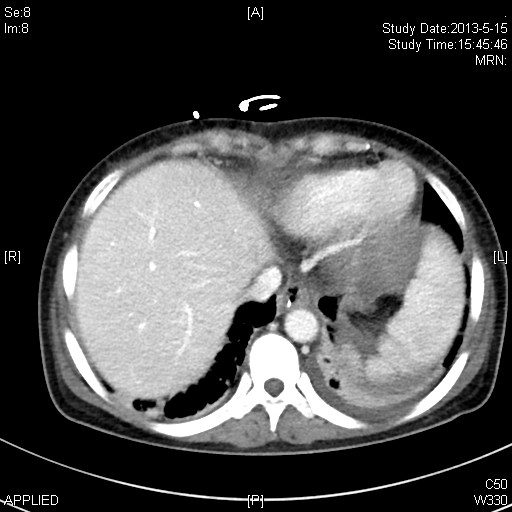

Supplement: Additional file 6: — Variations in phylogenetic distribution of 16S rDNA sequences following fecal infusion. The diagrammatic phylogenetic tree presents a summary of the rRNA sequences obtained from DGGE bands in this study. Phyla are named to the left of the tree, and lower taxonomic levels are given to the right. The number in the clade is designated as the relative proportion (%) in the whole fecal microbiota. [file 13054_2015_738_MOESM6_ESM.zip › CT images/CT images at 22 days after the surgery/Im8.jpg]

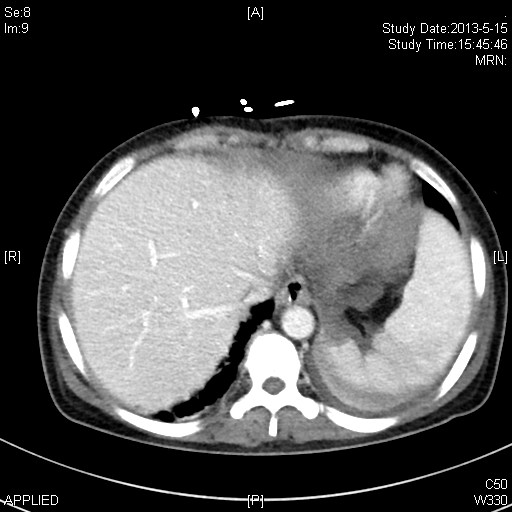

Supplement: Additional file 6: — Variations in phylogenetic distribution of 16S rDNA sequences following fecal infusion. The diagrammatic phylogenetic tree presents a summary of the rRNA sequences obtained from DGGE bands in this study. Phyla are named to the left of the tree, and lower taxonomic levels are given to the right. The number in the clade is designated as the relative proportion (%) in the whole fecal microbiota. [file 13054_2015_738_MOESM6_ESM.zip › CT images/CT images at 22 days after the surgery/Im9.jpg]

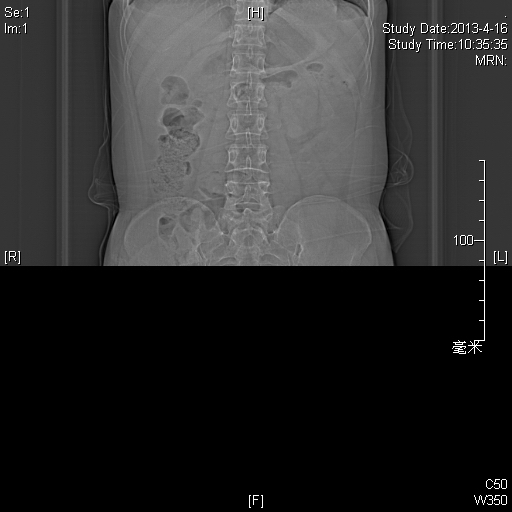

Supplement: Additional file 6: — Variations in phylogenetic distribution of 16S rDNA sequences following fecal infusion. The diagrammatic phylogenetic tree presents a summary of the rRNA sequences obtained from DGGE bands in this study. Phyla are named to the left of the tree, and lower taxonomic levels are given to the right. The number in the clade is designated as the relative proportion (%) in the whole fecal microbiota. [file 13054_2015_738_MOESM6_ESM.zip › CT images/CT images at 7 days before the surgery/Im01.jpg]

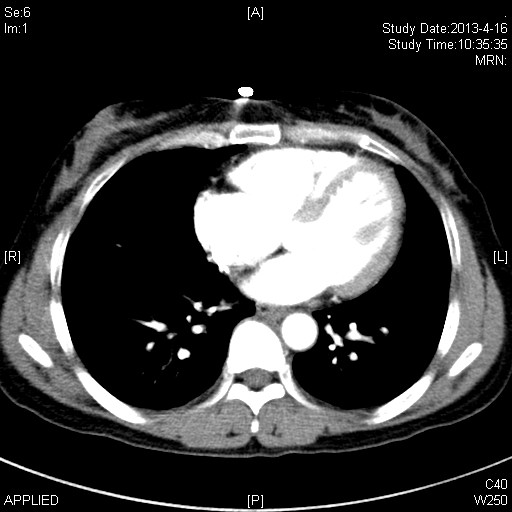

Supplement: Additional file 6: — Variations in phylogenetic distribution of 16S rDNA sequences following fecal infusion. The diagrammatic phylogenetic tree presents a summary of the rRNA sequences obtained from DGGE bands in this study. Phyla are named to the left of the tree, and lower taxonomic levels are given to the right. The number in the clade is designated as the relative proportion (%) in the whole fecal microbiota. [file 13054_2015_738_MOESM6_ESM.zip › CT images/CT images at 7 days before the surgery/Im1.jpg]

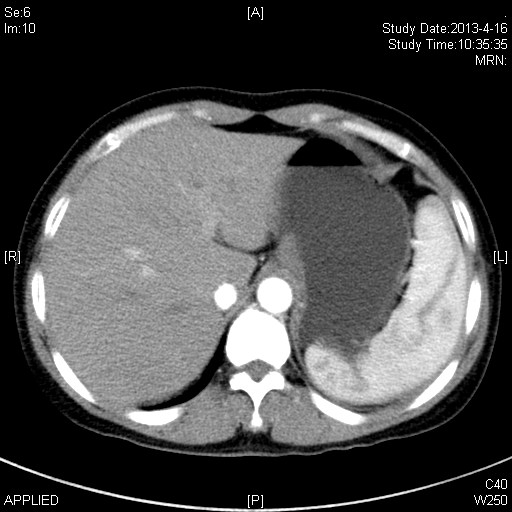

Supplement: Additional file 6: — Variations in phylogenetic distribution of 16S rDNA sequences following fecal infusion. The diagrammatic phylogenetic tree presents a summary of the rRNA sequences obtained from DGGE bands in this study. Phyla are named to the left of the tree, and lower taxonomic levels are given to the right. The number in the clade is designated as the relative proportion (%) in the whole fecal microbiota. [file 13054_2015_738_MOESM6_ESM.zip › CT images/CT images at 7 days before the surgery/Im10.jpg]

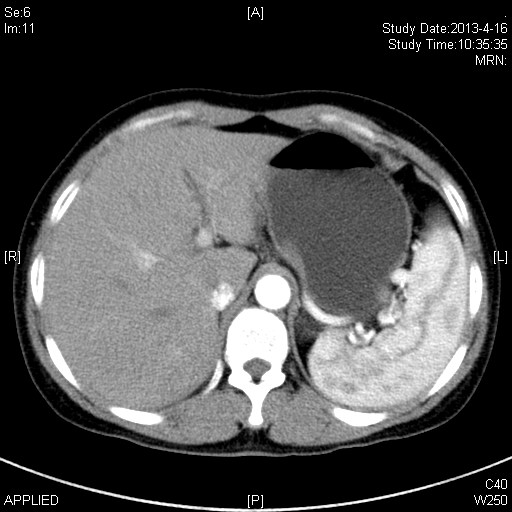

Supplement: Additional file 6: — Variations in phylogenetic distribution of 16S rDNA sequences following fecal infusion. The diagrammatic phylogenetic tree presents a summary of the rRNA sequences obtained from DGGE bands in this study. Phyla are named to the left of the tree, and lower taxonomic levels are given to the right. The number in the clade is designated as the relative proportion (%) in the whole fecal microbiota. [file 13054_2015_738_MOESM6_ESM.zip › CT images/CT images at 7 days before the surgery/Im11.jpg]

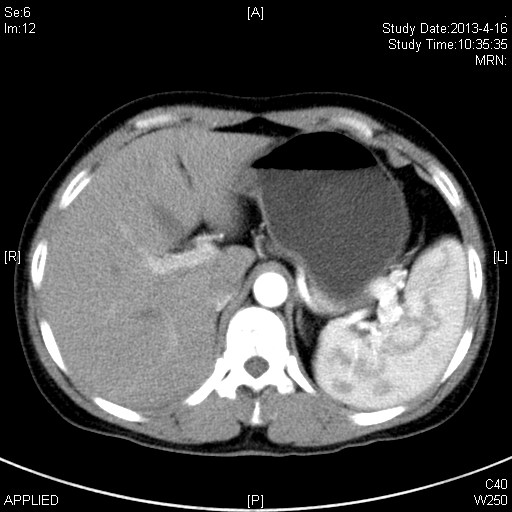

Supplement: Additional file 6: — Variations in phylogenetic distribution of 16S rDNA sequences following fecal infusion. The diagrammatic phylogenetic tree presents a summary of the rRNA sequences obtained from DGGE bands in this study. Phyla are named to the left of the tree, and lower taxonomic levels are given to the right. The number in the clade is designated as the relative proportion (%) in the whole fecal microbiota. [file 13054_2015_738_MOESM6_ESM.zip › CT images/CT images at 7 days before the surgery/Im12.jpg]

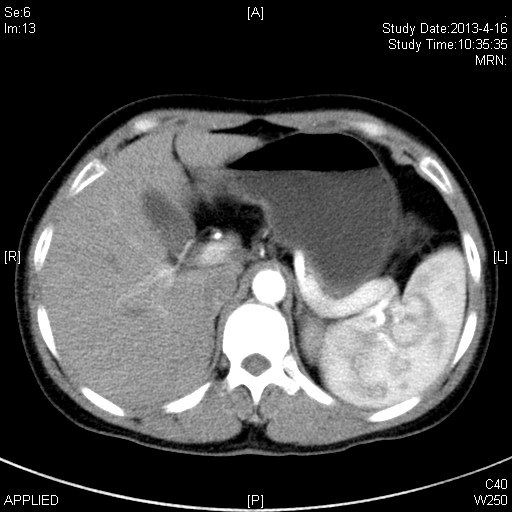

Supplement: Additional file 6: — Variations in phylogenetic distribution of 16S rDNA sequences following fecal infusion. The diagrammatic phylogenetic tree presents a summary of the rRNA sequences obtained from DGGE bands in this study. Phyla are named to the left of the tree, and lower taxonomic levels are given to the right. The number in the clade is designated as the relative proportion (%) in the whole fecal microbiota. [file 13054_2015_738_MOESM6_ESM.zip › CT images/CT images at 7 days before the surgery/Im13.jpg]

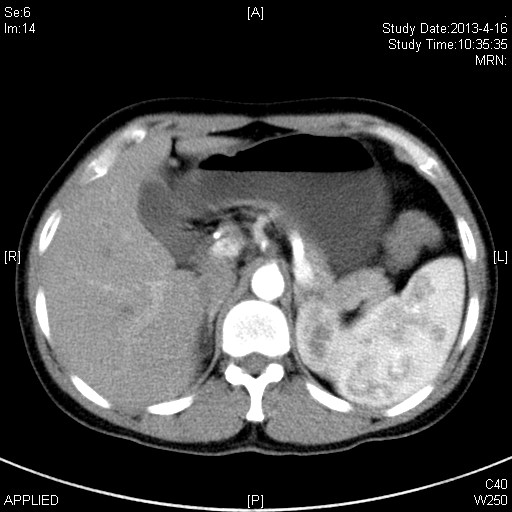

Supplement: Additional file 6: — Variations in phylogenetic distribution of 16S rDNA sequences following fecal infusion. The diagrammatic phylogenetic tree presents a summary of the rRNA sequences obtained from DGGE bands in this study. Phyla are named to the left of the tree, and lower taxonomic levels are given to the right. The number in the clade is designated as the relative proportion (%) in the whole fecal microbiota. [file 13054_2015_738_MOESM6_ESM.zip › CT images/CT images at 7 days before the surgery/Im14.jpg]

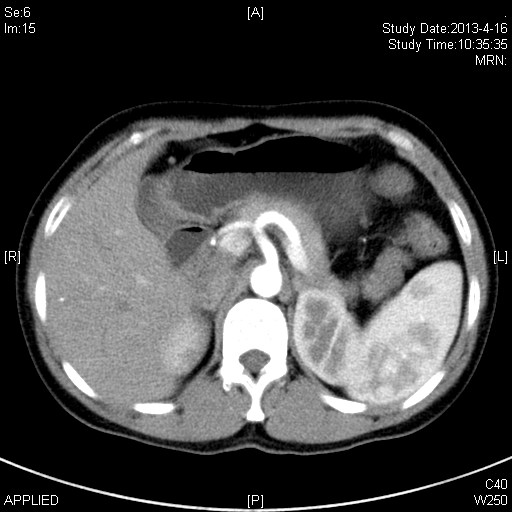

Supplement: Additional file 6: — Variations in phylogenetic distribution of 16S rDNA sequences following fecal infusion. The diagrammatic phylogenetic tree presents a summary of the rRNA sequences obtained from DGGE bands in this study. Phyla are named to the left of the tree, and lower taxonomic levels are given to the right. The number in the clade is designated as the relative proportion (%) in the whole fecal microbiota. [file 13054_2015_738_MOESM6_ESM.zip › CT images/CT images at 7 days before the surgery/Im15.jpg]

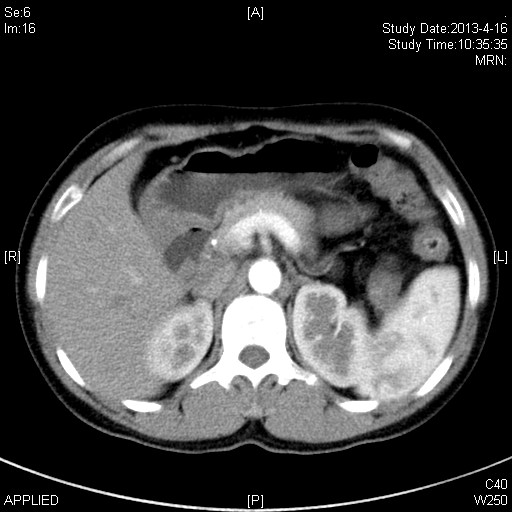

Supplement: Additional file 6: — Variations in phylogenetic distribution of 16S rDNA sequences following fecal infusion. The diagrammatic phylogenetic tree presents a summary of the rRNA sequences obtained from DGGE bands in this study. Phyla are named to the left of the tree, and lower taxonomic levels are given to the right. The number in the clade is designated as the relative proportion (%) in the whole fecal microbiota. [file 13054_2015_738_MOESM6_ESM.zip › CT images/CT images at 7 days before the surgery/Im16.jpg]

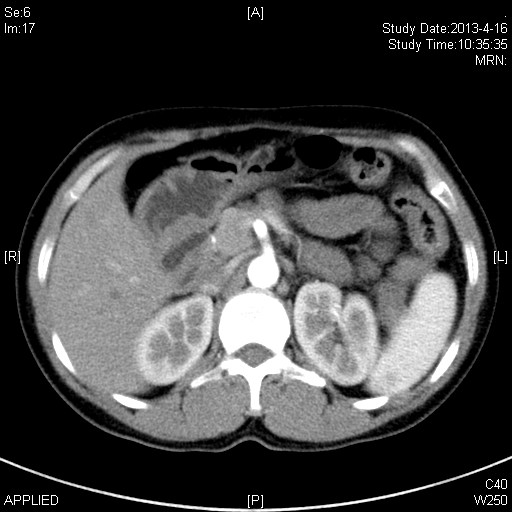

Supplement: Additional file 6: — Variations in phylogenetic distribution of 16S rDNA sequences following fecal infusion. The diagrammatic phylogenetic tree presents a summary of the rRNA sequences obtained from DGGE bands in this study. Phyla are named to the left of the tree, and lower taxonomic levels are given to the right. The number in the clade is designated as the relative proportion (%) in the whole fecal microbiota. [file 13054_2015_738_MOESM6_ESM.zip › CT images/CT images at 7 days before the surgery/Im17.jpg]

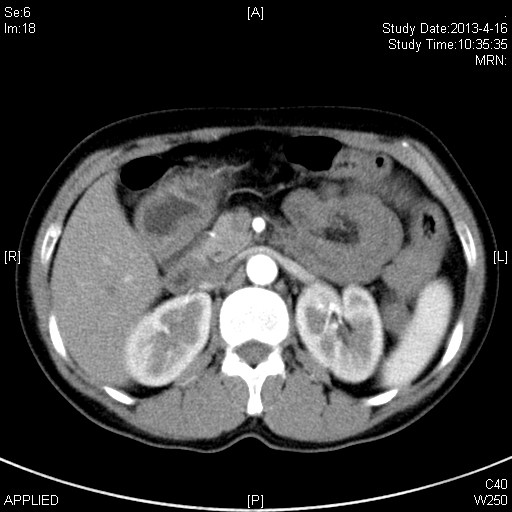

Supplement: Additional file 6: — Variations in phylogenetic distribution of 16S rDNA sequences following fecal infusion. The diagrammatic phylogenetic tree presents a summary of the rRNA sequences obtained from DGGE bands in this study. Phyla are named to the left of the tree, and lower taxonomic levels are given to the right. The number in the clade is designated as the relative proportion (%) in the whole fecal microbiota. [file 13054_2015_738_MOESM6_ESM.zip › CT images/CT images at 7 days before the surgery/Im18.jpg]

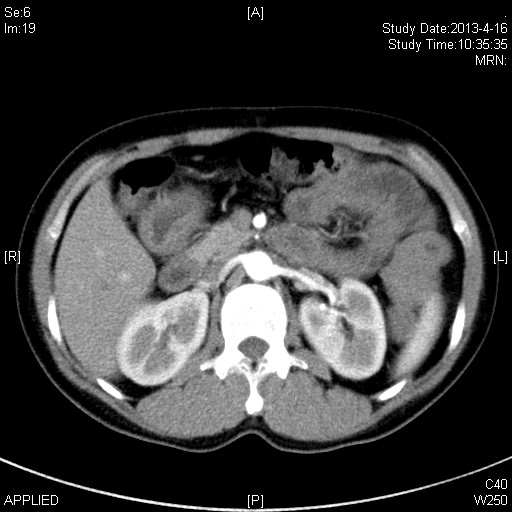

Supplement: Additional file 6: — Variations in phylogenetic distribution of 16S rDNA sequences following fecal infusion. The diagrammatic phylogenetic tree presents a summary of the rRNA sequences obtained from DGGE bands in this study. Phyla are named to the left of the tree, and lower taxonomic levels are given to the right. The number in the clade is designated as the relative proportion (%) in the whole fecal microbiota. [file 13054_2015_738_MOESM6_ESM.zip › CT images/CT images at 7 days before the surgery/Im19.jpg]

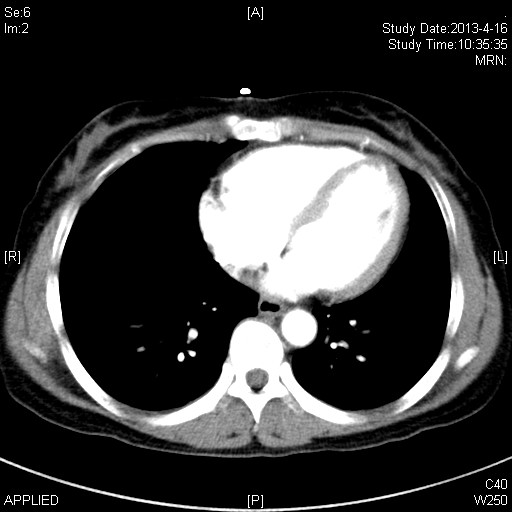

Supplement: Additional file 6: — Variations in phylogenetic distribution of 16S rDNA sequences following fecal infusion. The diagrammatic phylogenetic tree presents a summary of the rRNA sequences obtained from DGGE bands in this study. Phyla are named to the left of the tree, and lower taxonomic levels are given to the right. The number in the clade is designated as the relative proportion (%) in the whole fecal microbiota. [file 13054_2015_738_MOESM6_ESM.zip › CT images/CT images at 7 days before the surgery/Im2.jpg]

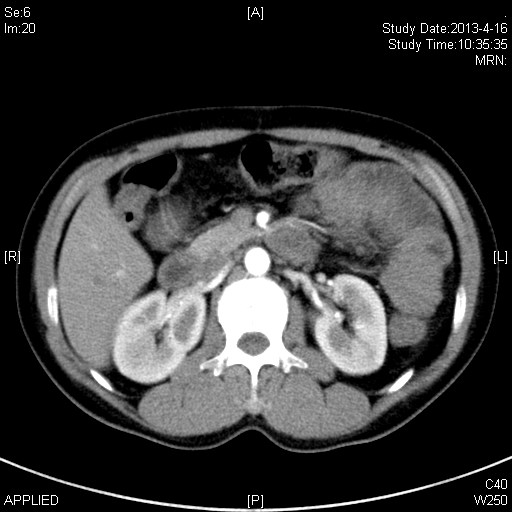

Supplement: Additional file 6: — Variations in phylogenetic distribution of 16S rDNA sequences following fecal infusion. The diagrammatic phylogenetic tree presents a summary of the rRNA sequences obtained from DGGE bands in this study. Phyla are named to the left of the tree, and lower taxonomic levels are given to the right. The number in the clade is designated as the relative proportion (%) in the whole fecal microbiota. [file 13054_2015_738_MOESM6_ESM.zip › CT images/CT images at 7 days before the surgery/Im20.jpg]

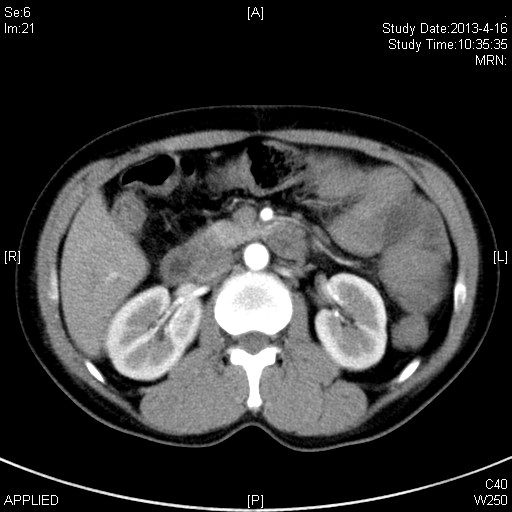

Supplement: Additional file 6: — Variations in phylogenetic distribution of 16S rDNA sequences following fecal infusion. The diagrammatic phylogenetic tree presents a summary of the rRNA sequences obtained from DGGE bands in this study. Phyla are named to the left of the tree, and lower taxonomic levels are given to the right. The number in the clade is designated as the relative proportion (%) in the whole fecal microbiota. [file 13054_2015_738_MOESM6_ESM.zip › CT images/CT images at 7 days before the surgery/Im21.jpg]

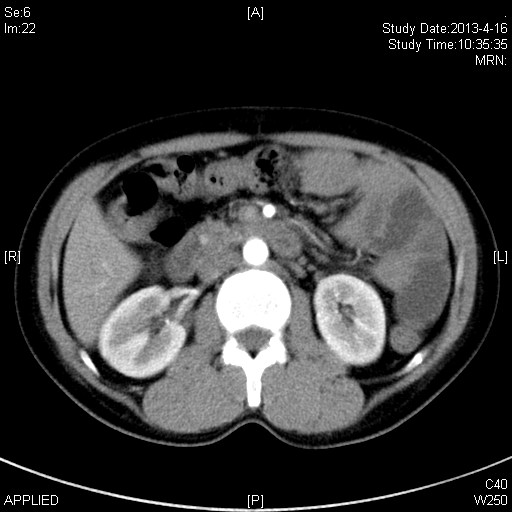

Supplement: Additional file 6: — Variations in phylogenetic distribution of 16S rDNA sequences following fecal infusion. The diagrammatic phylogenetic tree presents a summary of the rRNA sequences obtained from DGGE bands in this study. Phyla are named to the left of the tree, and lower taxonomic levels are given to the right. The number in the clade is designated as the relative proportion (%) in the whole fecal microbiota. [file 13054_2015_738_MOESM6_ESM.zip › CT images/CT images at 7 days before the surgery/Im22.jpg]

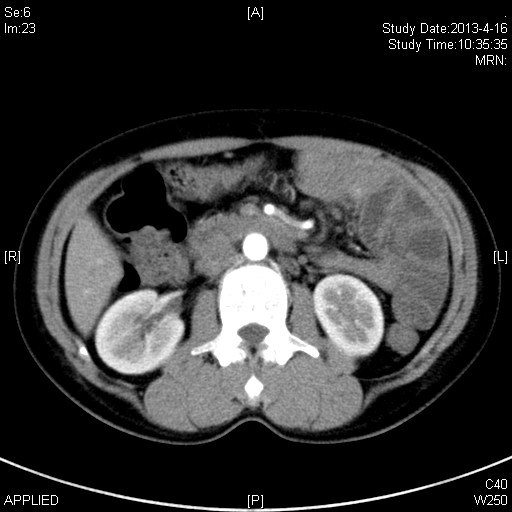

Supplement: Additional file 6: — Variations in phylogenetic distribution of 16S rDNA sequences following fecal infusion. The diagrammatic phylogenetic tree presents a summary of the rRNA sequences obtained from DGGE bands in this study. Phyla are named to the left of the tree, and lower taxonomic levels are given to the right. The number in the clade is designated as the relative proportion (%) in the whole fecal microbiota. [file 13054_2015_738_MOESM6_ESM.zip › CT images/CT images at 7 days before the surgery/Im23.jpg]

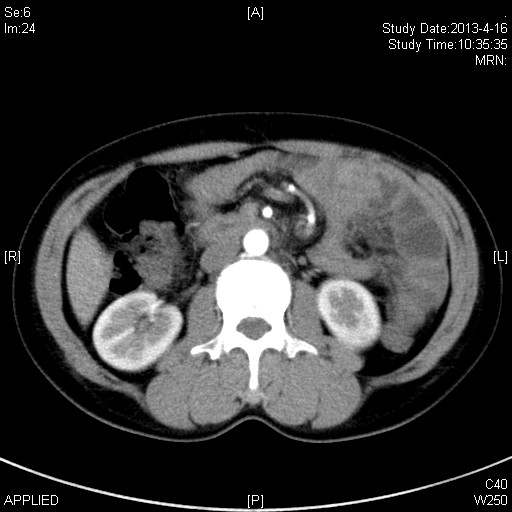

Supplement: Additional file 6: — Variations in phylogenetic distribution of 16S rDNA sequences following fecal infusion. The diagrammatic phylogenetic tree presents a summary of the rRNA sequences obtained from DGGE bands in this study. Phyla are named to the left of the tree, and lower taxonomic levels are given to the right. The number in the clade is designated as the relative proportion (%) in the whole fecal microbiota. [file 13054_2015_738_MOESM6_ESM.zip › CT images/CT images at 7 days before the surgery/Im24.jpg]

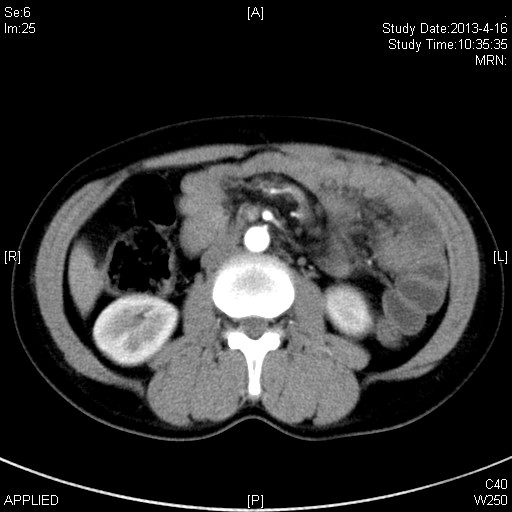

Supplement: Additional file 6: — Variations in phylogenetic distribution of 16S rDNA sequences following fecal infusion. The diagrammatic phylogenetic tree presents a summary of the rRNA sequences obtained from DGGE bands in this study. Phyla are named to the left of the tree, and lower taxonomic levels are given to the right. The number in the clade is designated as the relative proportion (%) in the whole fecal microbiota. [file 13054_2015_738_MOESM6_ESM.zip › CT images/CT images at 7 days before the surgery/Im25.jpg]

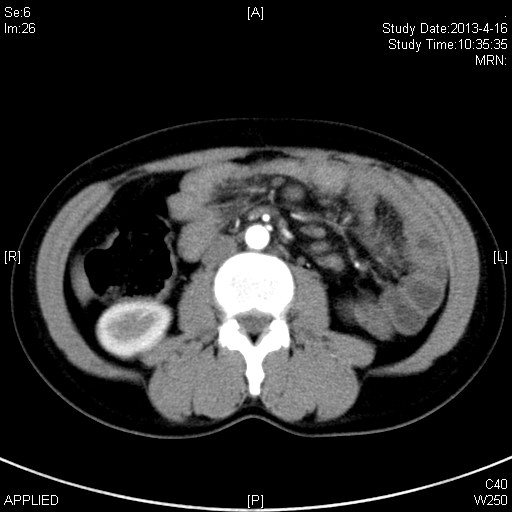

Supplement: Additional file 6: — Variations in phylogenetic distribution of 16S rDNA sequences following fecal infusion. The diagrammatic phylogenetic tree presents a summary of the rRNA sequences obtained from DGGE bands in this study. Phyla are named to the left of the tree, and lower taxonomic levels are given to the right. The number in the clade is designated as the relative proportion (%) in the whole fecal microbiota. [file 13054_2015_738_MOESM6_ESM.zip › CT images/CT images at 7 days before the surgery/Im26.jpg]

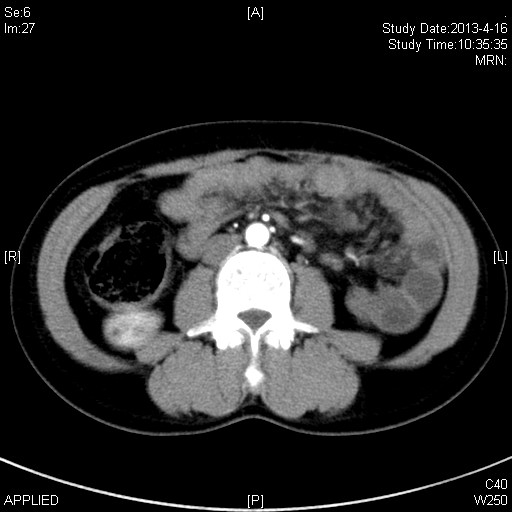

Supplement: Additional file 6: — Variations in phylogenetic distribution of 16S rDNA sequences following fecal infusion. The diagrammatic phylogenetic tree presents a summary of the rRNA sequences obtained from DGGE bands in this study. Phyla are named to the left of the tree, and lower taxonomic levels are given to the right. The number in the clade is designated as the relative proportion (%) in the whole fecal microbiota. [file 13054_2015_738_MOESM6_ESM.zip › CT images/CT images at 7 days before the surgery/Im27.jpg]

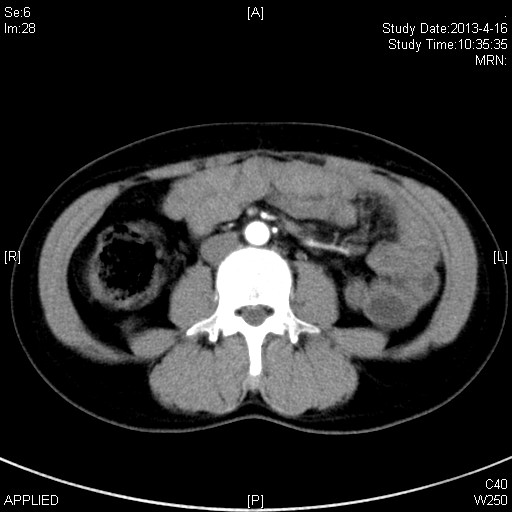

Supplement: Additional file 6: — Variations in phylogenetic distribution of 16S rDNA sequences following fecal infusion. The diagrammatic phylogenetic tree presents a summary of the rRNA sequences obtained from DGGE bands in this study. Phyla are named to the left of the tree, and lower taxonomic levels are given to the right. The number in the clade is designated as the relative proportion (%) in the whole fecal microbiota. [file 13054_2015_738_MOESM6_ESM.zip › CT images/CT images at 7 days before the surgery/Im28.jpg]

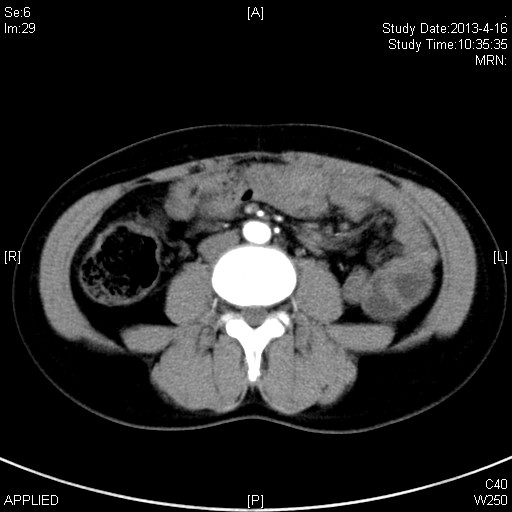

Supplement: Additional file 6: — Variations in phylogenetic distribution of 16S rDNA sequences following fecal infusion. The diagrammatic phylogenetic tree presents a summary of the rRNA sequences obtained from DGGE bands in this study. Phyla are named to the left of the tree, and lower taxonomic levels are given to the right. The number in the clade is designated as the relative proportion (%) in the whole fecal microbiota. [file 13054_2015_738_MOESM6_ESM.zip › CT images/CT images at 7 days before the surgery/Im29.jpg]

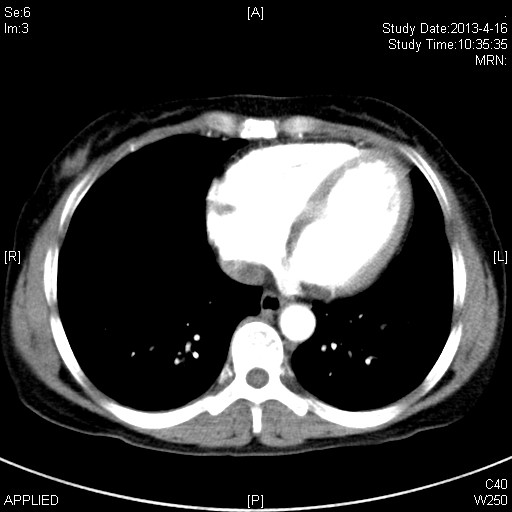

Supplement: Additional file 6: — Variations in phylogenetic distribution of 16S rDNA sequences following fecal infusion. The diagrammatic phylogenetic tree presents a summary of the rRNA sequences obtained from DGGE bands in this study. Phyla are named to the left of the tree, and lower taxonomic levels are given to the right. The number in the clade is designated as the relative proportion (%) in the whole fecal microbiota. [file 13054_2015_738_MOESM6_ESM.zip › CT images/CT images at 7 days before the surgery/Im3.jpg]

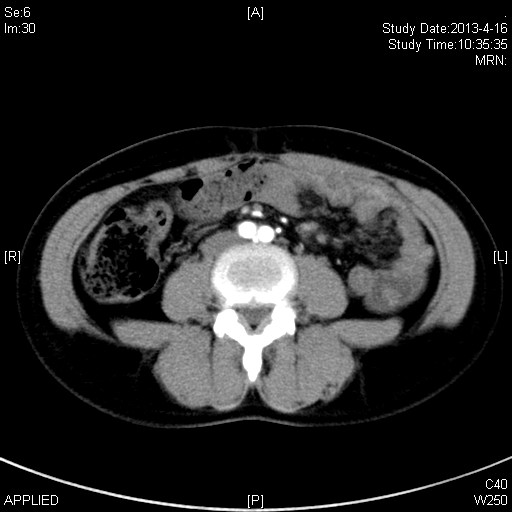

Supplement: Additional file 6: — Variations in phylogenetic distribution of 16S rDNA sequences following fecal infusion. The diagrammatic phylogenetic tree presents a summary of the rRNA sequences obtained from DGGE bands in this study. Phyla are named to the left of the tree, and lower taxonomic levels are given to the right. The number in the clade is designated as the relative proportion (%) in the whole fecal microbiota. [file 13054_2015_738_MOESM6_ESM.zip › CT images/CT images at 7 days before the surgery/Im30.jpg]

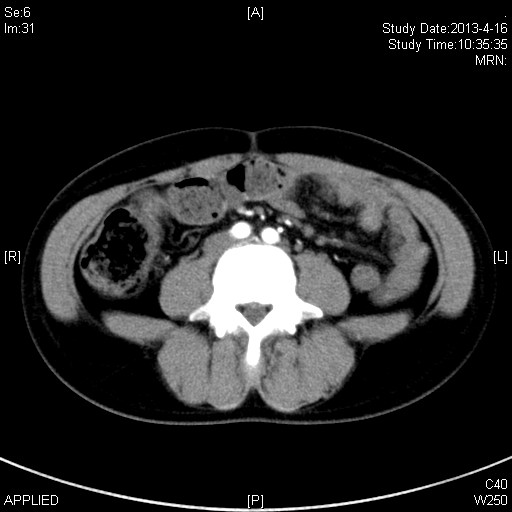

Supplement: Additional file 6: — Variations in phylogenetic distribution of 16S rDNA sequences following fecal infusion. The diagrammatic phylogenetic tree presents a summary of the rRNA sequences obtained from DGGE bands in this study. Phyla are named to the left of the tree, and lower taxonomic levels are given to the right. The number in the clade is designated as the relative proportion (%) in the whole fecal microbiota. [file 13054_2015_738_MOESM6_ESM.zip › CT images/CT images at 7 days before the surgery/Im31.jpg]

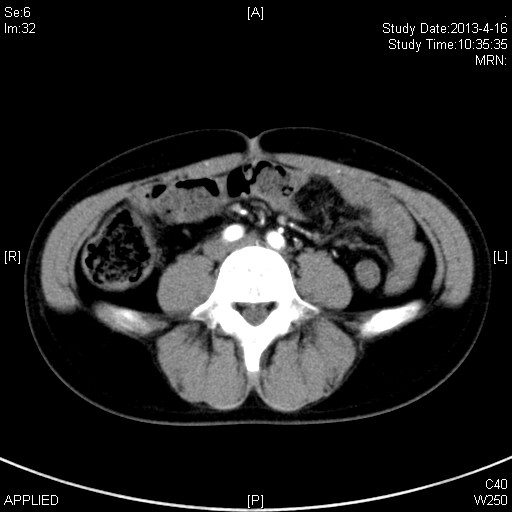

Supplement: Additional file 6: — Variations in phylogenetic distribution of 16S rDNA sequences following fecal infusion. The diagrammatic phylogenetic tree presents a summary of the rRNA sequences obtained from DGGE bands in this study. Phyla are named to the left of the tree, and lower taxonomic levels are given to the right. The number in the clade is designated as the relative proportion (%) in the whole fecal microbiota. [file 13054_2015_738_MOESM6_ESM.zip › CT images/CT images at 7 days before the surgery/Im32.jpg]

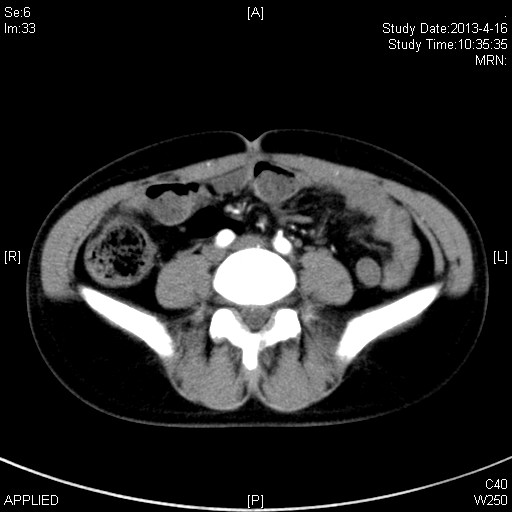

Supplement: Additional file 6: — Variations in phylogenetic distribution of 16S rDNA sequences following fecal infusion. The diagrammatic phylogenetic tree presents a summary of the rRNA sequences obtained from DGGE bands in this study. Phyla are named to the left of the tree, and lower taxonomic levels are given to the right. The number in the clade is designated as the relative proportion (%) in the whole fecal microbiota. [file 13054_2015_738_MOESM6_ESM.zip › CT images/CT images at 7 days before the surgery/Im33.jpg]

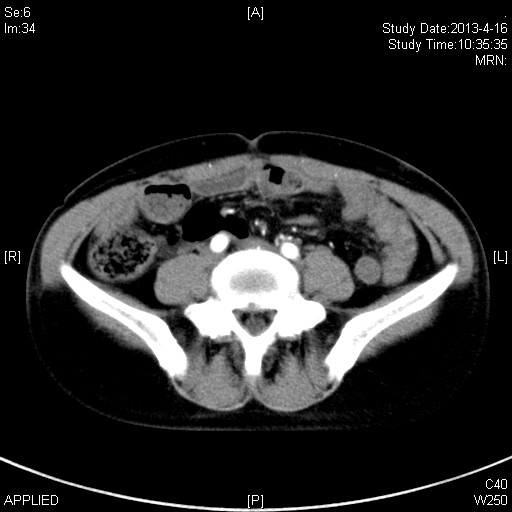

Supplement: Additional file 6: — Variations in phylogenetic distribution of 16S rDNA sequences following fecal infusion. The diagrammatic phylogenetic tree presents a summary of the rRNA sequences obtained from DGGE bands in this study. Phyla are named to the left of the tree, and lower taxonomic levels are given to the right. The number in the clade is designated as the relative proportion (%) in the whole fecal microbiota. [file 13054_2015_738_MOESM6_ESM.zip › CT images/CT images at 7 days before the surgery/Im34.jpg]

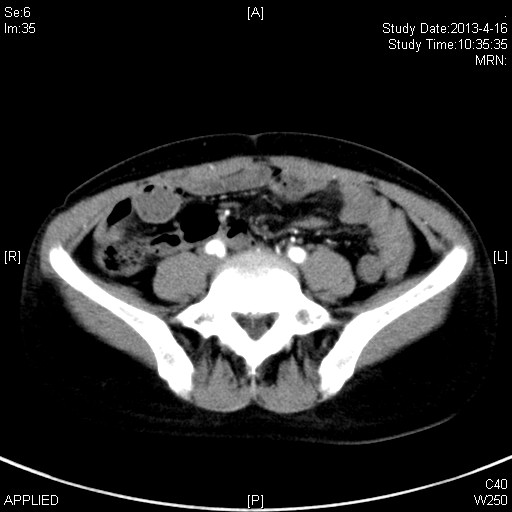

Supplement: Additional file 6: — Variations in phylogenetic distribution of 16S rDNA sequences following fecal infusion. The diagrammatic phylogenetic tree presents a summary of the rRNA sequences obtained from DGGE bands in this study. Phyla are named to the left of the tree, and lower taxonomic levels are given to the right. The number in the clade is designated as the relative proportion (%) in the whole fecal microbiota. [file 13054_2015_738_MOESM6_ESM.zip › CT images/CT images at 7 days before the surgery/Im35.jpg]

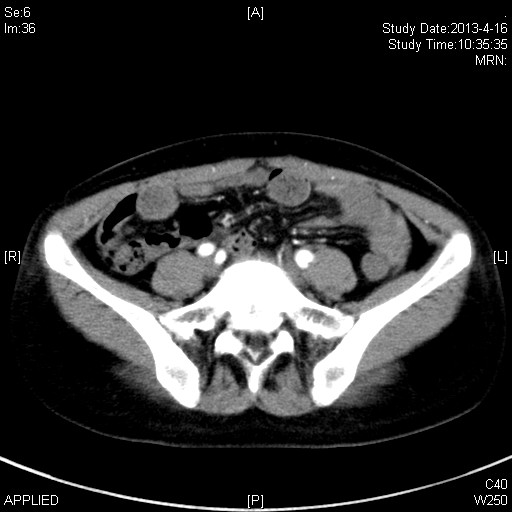

Supplement: Additional file 6: — Variations in phylogenetic distribution of 16S rDNA sequences following fecal infusion. The diagrammatic phylogenetic tree presents a summary of the rRNA sequences obtained from DGGE bands in this study. Phyla are named to the left of the tree, and lower taxonomic levels are given to the right. The number in the clade is designated as the relative proportion (%) in the whole fecal microbiota. [file 13054_2015_738_MOESM6_ESM.zip › CT images/CT images at 7 days before the surgery/Im36.jpg]

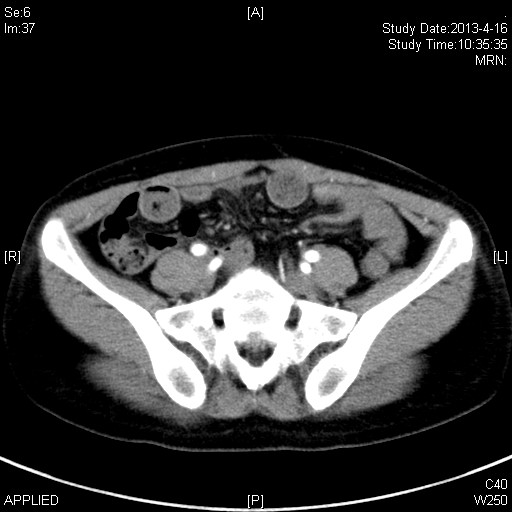

Supplement: Additional file 6: — Variations in phylogenetic distribution of 16S rDNA sequences following fecal infusion. The diagrammatic phylogenetic tree presents a summary of the rRNA sequences obtained from DGGE bands in this study. Phyla are named to the left of the tree, and lower taxonomic levels are given to the right. The number in the clade is designated as the relative proportion (%) in the whole fecal microbiota. [file 13054_2015_738_MOESM6_ESM.zip › CT images/CT images at 7 days before the surgery/Im37.jpg]

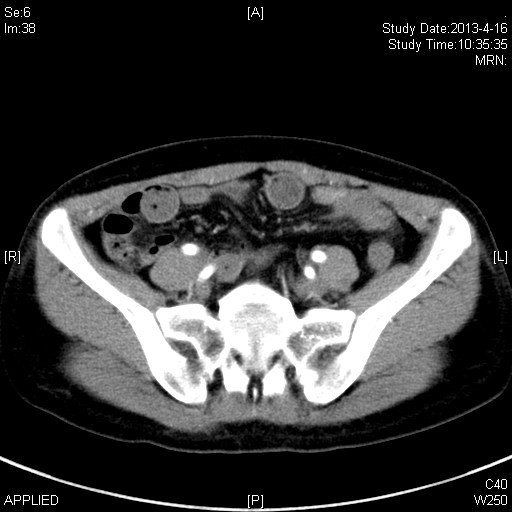

Supplement: Additional file 6: — Variations in phylogenetic distribution of 16S rDNA sequences following fecal infusion. The diagrammatic phylogenetic tree presents a summary of the rRNA sequences obtained from DGGE bands in this study. Phyla are named to the left of the tree, and lower taxonomic levels are given to the right. The number in the clade is designated as the relative proportion (%) in the whole fecal microbiota. [file 13054_2015_738_MOESM6_ESM.zip › CT images/CT images at 7 days before the surgery/Im38.jpg]

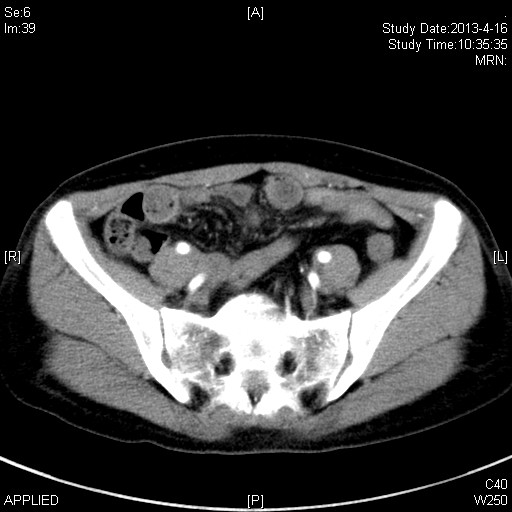

Supplement: Additional file 6: — Variations in phylogenetic distribution of 16S rDNA sequences following fecal infusion. The diagrammatic phylogenetic tree presents a summary of the rRNA sequences obtained from DGGE bands in this study. Phyla are named to the left of the tree, and lower taxonomic levels are given to the right. The number in the clade is designated as the relative proportion (%) in the whole fecal microbiota. [file 13054_2015_738_MOESM6_ESM.zip › CT images/CT images at 7 days before the surgery/Im39.jpg]

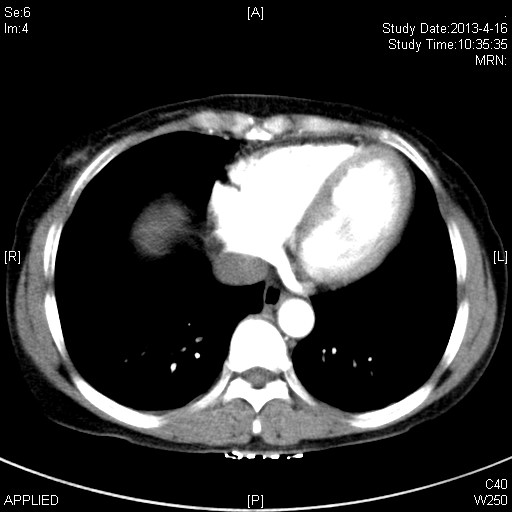

Supplement: Additional file 6: — Variations in phylogenetic distribution of 16S rDNA sequences following fecal infusion. The diagrammatic phylogenetic tree presents a summary of the rRNA sequences obtained from DGGE bands in this study. Phyla are named to the left of the tree, and lower taxonomic levels are given to the right. The number in the clade is designated as the relative proportion (%) in the whole fecal microbiota. [file 13054_2015_738_MOESM6_ESM.zip › CT images/CT images at 7 days before the surgery/Im4.jpg]

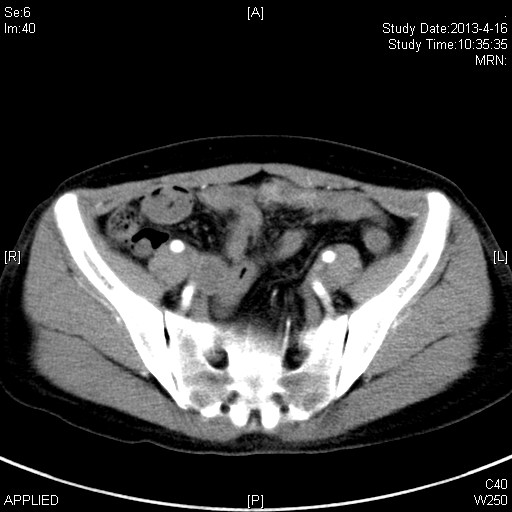

Supplement: Additional file 6: — Variations in phylogenetic distribution of 16S rDNA sequences following fecal infusion. The diagrammatic phylogenetic tree presents a summary of the rRNA sequences obtained from DGGE bands in this study. Phyla are named to the left of the tree, and lower taxonomic levels are given to the right. The number in the clade is designated as the relative proportion (%) in the whole fecal microbiota. [file 13054_2015_738_MOESM6_ESM.zip › CT images/CT images at 7 days before the surgery/Im40.jpg]

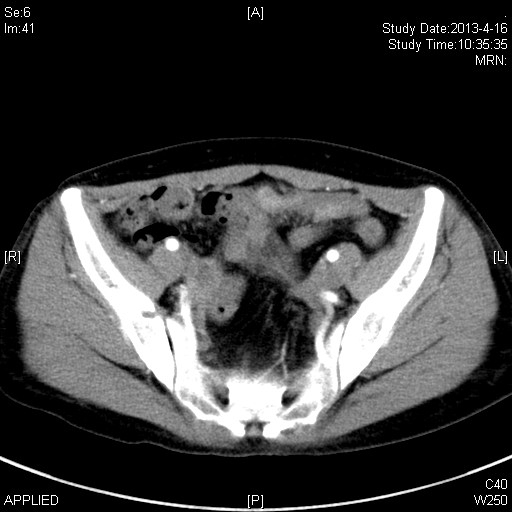

Supplement: Additional file 6: — Variations in phylogenetic distribution of 16S rDNA sequences following fecal infusion. The diagrammatic phylogenetic tree presents a summary of the rRNA sequences obtained from DGGE bands in this study. Phyla are named to the left of the tree, and lower taxonomic levels are given to the right. The number in the clade is designated as the relative proportion (%) in the whole fecal microbiota. [file 13054_2015_738_MOESM6_ESM.zip › CT images/CT images at 7 days before the surgery/Im41.jpg]

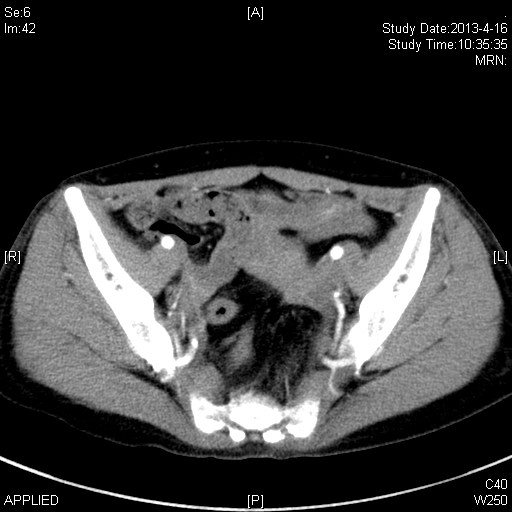

Supplement: Additional file 6: — Variations in phylogenetic distribution of 16S rDNA sequences following fecal infusion. The diagrammatic phylogenetic tree presents a summary of the rRNA sequences obtained from DGGE bands in this study. Phyla are named to the left of the tree, and lower taxonomic levels are given to the right. The number in the clade is designated as the relative proportion (%) in the whole fecal microbiota. [file 13054_2015_738_MOESM6_ESM.zip › CT images/CT images at 7 days before the surgery/Im42.jpg]

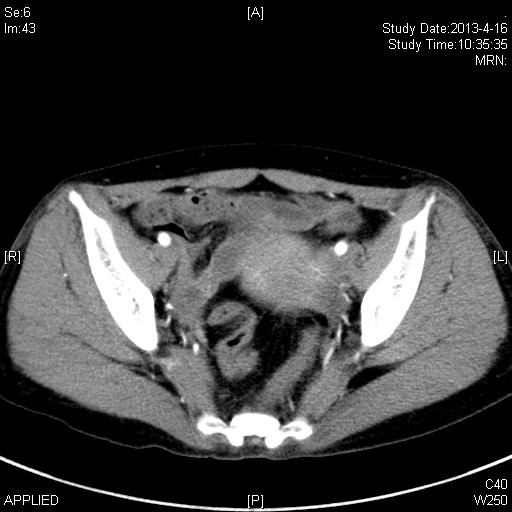

Supplement: Additional file 6: — Variations in phylogenetic distribution of 16S rDNA sequences following fecal infusion. The diagrammatic phylogenetic tree presents a summary of the rRNA sequences obtained from DGGE bands in this study. Phyla are named to the left of the tree, and lower taxonomic levels are given to the right. The number in the clade is designated as the relative proportion (%) in the whole fecal microbiota. [file 13054_2015_738_MOESM6_ESM.zip › CT images/CT images at 7 days before the surgery/Im43.jpg]

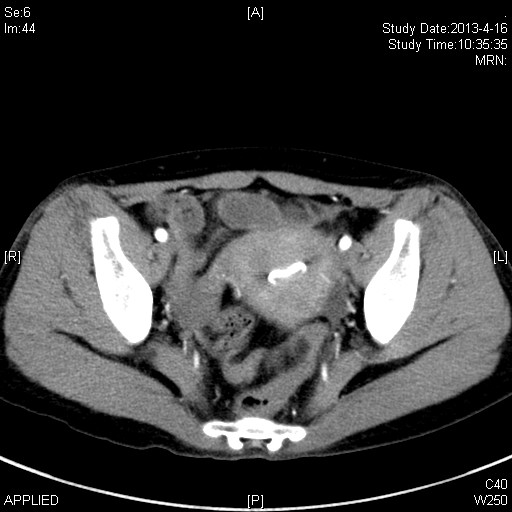

Supplement: Additional file 6: — Variations in phylogenetic distribution of 16S rDNA sequences following fecal infusion. The diagrammatic phylogenetic tree presents a summary of the rRNA sequences obtained from DGGE bands in this study. Phyla are named to the left of the tree, and lower taxonomic levels are given to the right. The number in the clade is designated as the relative proportion (%) in the whole fecal microbiota. [file 13054_2015_738_MOESM6_ESM.zip › CT images/CT images at 7 days before the surgery/Im44.jpg]

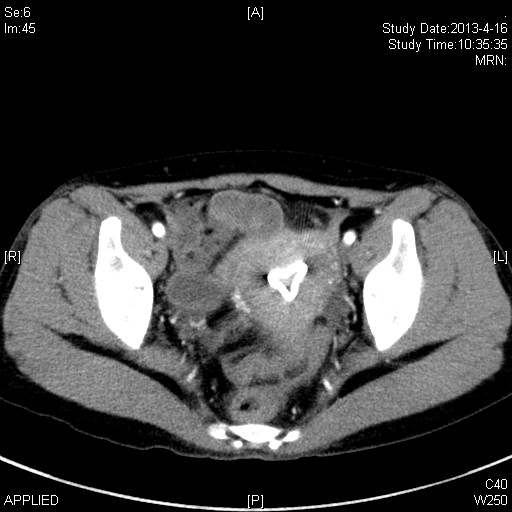

Supplement: Additional file 6: — Variations in phylogenetic distribution of 16S rDNA sequences following fecal infusion. The diagrammatic phylogenetic tree presents a summary of the rRNA sequences obtained from DGGE bands in this study. Phyla are named to the left of the tree, and lower taxonomic levels are given to the right. The number in the clade is designated as the relative proportion (%) in the whole fecal microbiota. [file 13054_2015_738_MOESM6_ESM.zip › CT images/CT images at 7 days before the surgery/Im45.jpg]

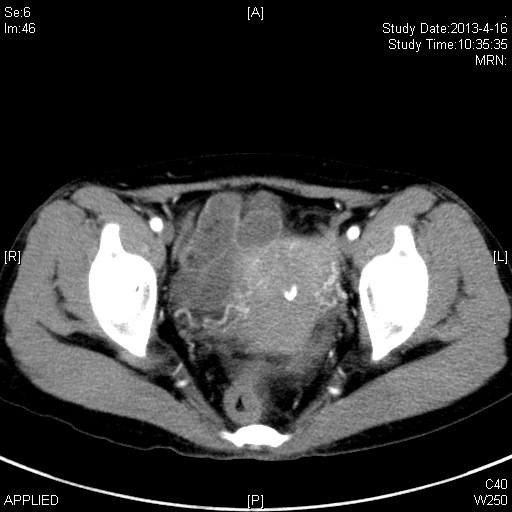

Supplement: Additional file 6: — Variations in phylogenetic distribution of 16S rDNA sequences following fecal infusion. The diagrammatic phylogenetic tree presents a summary of the rRNA sequences obtained from DGGE bands in this study. Phyla are named to the left of the tree, and lower taxonomic levels are given to the right. The number in the clade is designated as the relative proportion (%) in the whole fecal microbiota. [file 13054_2015_738_MOESM6_ESM.zip › CT images/CT images at 7 days before the surgery/Im46.jpg]

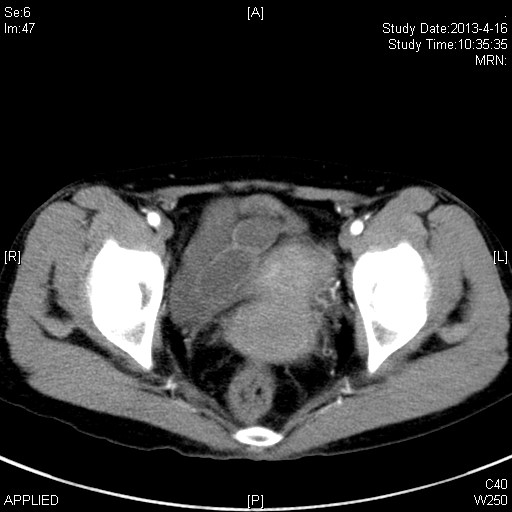

Supplement: Additional file 6: — Variations in phylogenetic distribution of 16S rDNA sequences following fecal infusion. The diagrammatic phylogenetic tree presents a summary of the rRNA sequences obtained from DGGE bands in this study. Phyla are named to the left of the tree, and lower taxonomic levels are given to the right. The number in the clade is designated as the relative proportion (%) in the whole fecal microbiota. [file 13054_2015_738_MOESM6_ESM.zip › CT images/CT images at 7 days before the surgery/Im47.jpg]

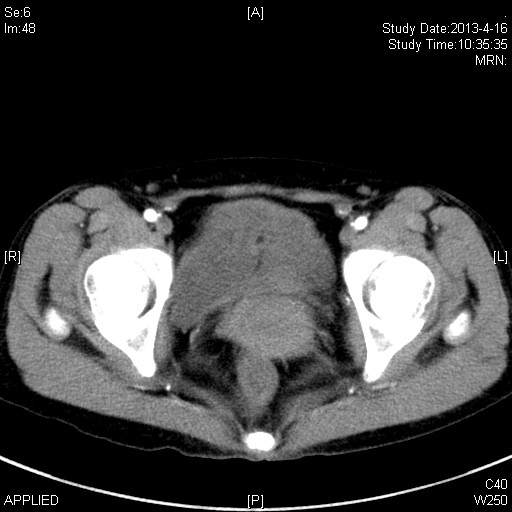

Supplement: Additional file 6: — Variations in phylogenetic distribution of 16S rDNA sequences following fecal infusion. The diagrammatic phylogenetic tree presents a summary of the rRNA sequences obtained from DGGE bands in this study. Phyla are named to the left of the tree, and lower taxonomic levels are given to the right. The number in the clade is designated as the relative proportion (%) in the whole fecal microbiota. [file 13054_2015_738_MOESM6_ESM.zip › CT images/CT images at 7 days before the surgery/Im48.jpg]

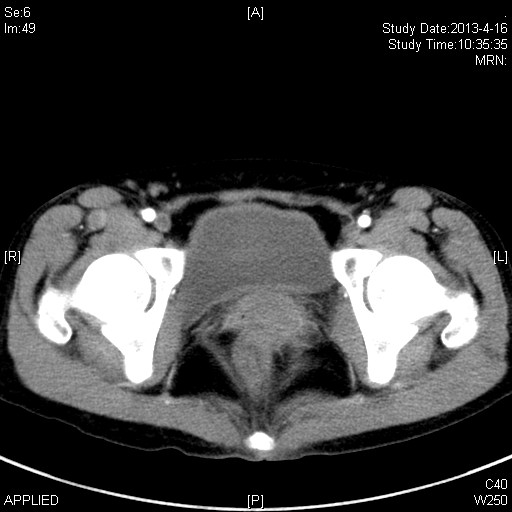

Supplement: Additional file 6: — Variations in phylogenetic distribution of 16S rDNA sequences following fecal infusion. The diagrammatic phylogenetic tree presents a summary of the rRNA sequences obtained from DGGE bands in this study. Phyla are named to the left of the tree, and lower taxonomic levels are given to the right. The number in the clade is designated as the relative proportion (%) in the whole fecal microbiota. [file 13054_2015_738_MOESM6_ESM.zip › CT images/CT images at 7 days before the surgery/Im49.jpg]
